# Practical classification of triple-negative breast cancer: intratumoral heterogeneity, mechanisms of drug resistance, and novel therapies

**DOI:** 10.1038/s41523-020-00197-2

**Published:** 2020-10-16

**Authors:** Antonio Marra, Dario Trapani, Giulia Viale, Carmen Criscitiello, Giuseppe Curigliano

**Affiliations:** 1 Division of Early Drug Development for Innovative Therapies, IEO, European Institute of Oncology IRCCS, Via Ripamonti, 435, 20141 Milan, Italy; 2 Department of Oncology and Haemato-Oncology, University of Milano, Via Festa del Perdono, 7, 20122 Milan, Italy

**Keywords:** Breast cancer, Breast cancer

## Abstract

Triple-negative breast cancer (TNBC) is not a unique disease, encompassing multiple entities with marked histopathological, transcriptomic and genomic heterogeneity. Despite several efforts, transcriptomic and genomic classifications have remained merely theoretic and most of the patients are being treated with chemotherapy. Driver alterations in potentially targetable genes, including PIK3CA and AKT, have been identified across TNBC subtypes, prompting the implementation of biomarker-driven therapeutic approaches. However, biomarker-based treatments as well as immune checkpoint inhibitor-based immunotherapy have provided contrasting and limited results so far. Accordingly, a better characterization of the genomic and immune contexture underpinning TNBC, as well as the translation of the lessons learnt in the metastatic disease to the early setting would improve patients’ outcomes. The application of multi-omics technologies, biocomputational algorithms, assays for minimal residual disease monitoring and novel clinical trial designs are strongly warranted to pave the way toward personalized anticancer treatment for patients with TNBC.

## Introduction

Triple-negative breast cancer (TNBC) refers to a subgroup of breast cancer (BC) defined by the lack of estrogen receptor (ER), progesterone receptor (PR), and human epidermal growth factor receptor 2 (HER2). Accounting for 15–20% of all BCs, TNBC is more prevalent in younger women, African and Hispanic descents and carriers of deleterious germline mutations in BC susceptibility genes^1^.

Pivotal microarray profiling studies categorized BC into five intrinsic subtypes^2^. Although the TNBC subgroup is considered a single entity based on immunohistochemistry (IHC), molecular profiling has revealed an unexpectedly level of heterogeneity. 50–75% of TNBCs have basal-like phenotype (BLBC), being characterized by the expression of genes of normal basal and myoepithelial cells^2,3^. Similarly, ∼80% of BLBCs are ER-negative and HER2-negative^4^. Even if the terms “TNBC” and “BLBC” are often used interchangeably, not all BLBCs determined by gene expression profiling (GEP) lack ER, PgR and HER2 and, conversely, not all TNBCs show a basal-like phenotype^5–8^. Moreover, another intrinsic subtype, namely claudin-low, was identified and characterized by a brisk stromal infiltration and expression of epithelial-to-mesenchymal-transition (EMT) and immune-response genes^9^, while the study that provided this information included a limited number of TNBC samples. Recently, claudin-low tumors were described as enriched in metaplastic histology variants, with lower levels of genomic instability, mutational burden, and targetable driver aberrations. Although the latter study identified claudin-low subtype to be associated with poor prognosis^10^, the heterogeneity among these studies requires further evidence to fully elucidate if the claudin low subtype can be intrinsically prognostic.

Further evidence demonstrated that TNBC is not a unique disease, encompassing multiple entities with pronounced histopathological, transcriptomic and genomic heterogeneity. Nonetheless, TNBC has been uniformly treated with chemotherapy. Exploiting TNBC diversity may help identifying new targetable pathways. Despite several efforts, these molecular classifications have remained merely theoretic and out of the clinical practice. Interestingly, potential targetable alterations have been identified across TNBC subtypes. Herein, we summarize the main evidence that defined transcriptomic and genomic heterogeneity of TNBC. Furthermore, we point out current and emerging biomarker-driven treatments for TNBC subtypes and describe the most common mechanisms of drug resistance for approved therapies in TNBC. Lastly, we discuss the challenges and possible future directions in the implementation of a biomarker-driven drug development in TNBC, potentially resulting in a practical classification of this BC subtype.

### Tumor heterogeneity of TNBC: an unsolved conundrum

TNBCs have several histology variants such as poor tumor differentiation, presence of metaplastic elements, medullary features, and stromal lymphocytic response^11–16^. In spite of this, TNBC spectrum also encompasses low-grade neoplasms. Despite being rare, these low-grade variants range from tumors with no or uncertain metastatic potential to invasive carcinomas. Several studies suggested that at least two subsets of low-grade TNBCs can be distinguished, including the low-grade TN breast neoplasia family (microglandular adenosis, atypical microglandular adenosis, and acinic cell carcinoma) and the salivary gland-like tumors of the breast^17^. Interestingly, these latter are characterized by salivary gland-like morphologic features and are often driven by specific genetic alterations, such as adenoid cystic and secretory carcinomas that are underpinned by the MYB-NFIB and ETV6-NTRK3 fusion genes, respectively^18,19^.

**Fig. 1:**
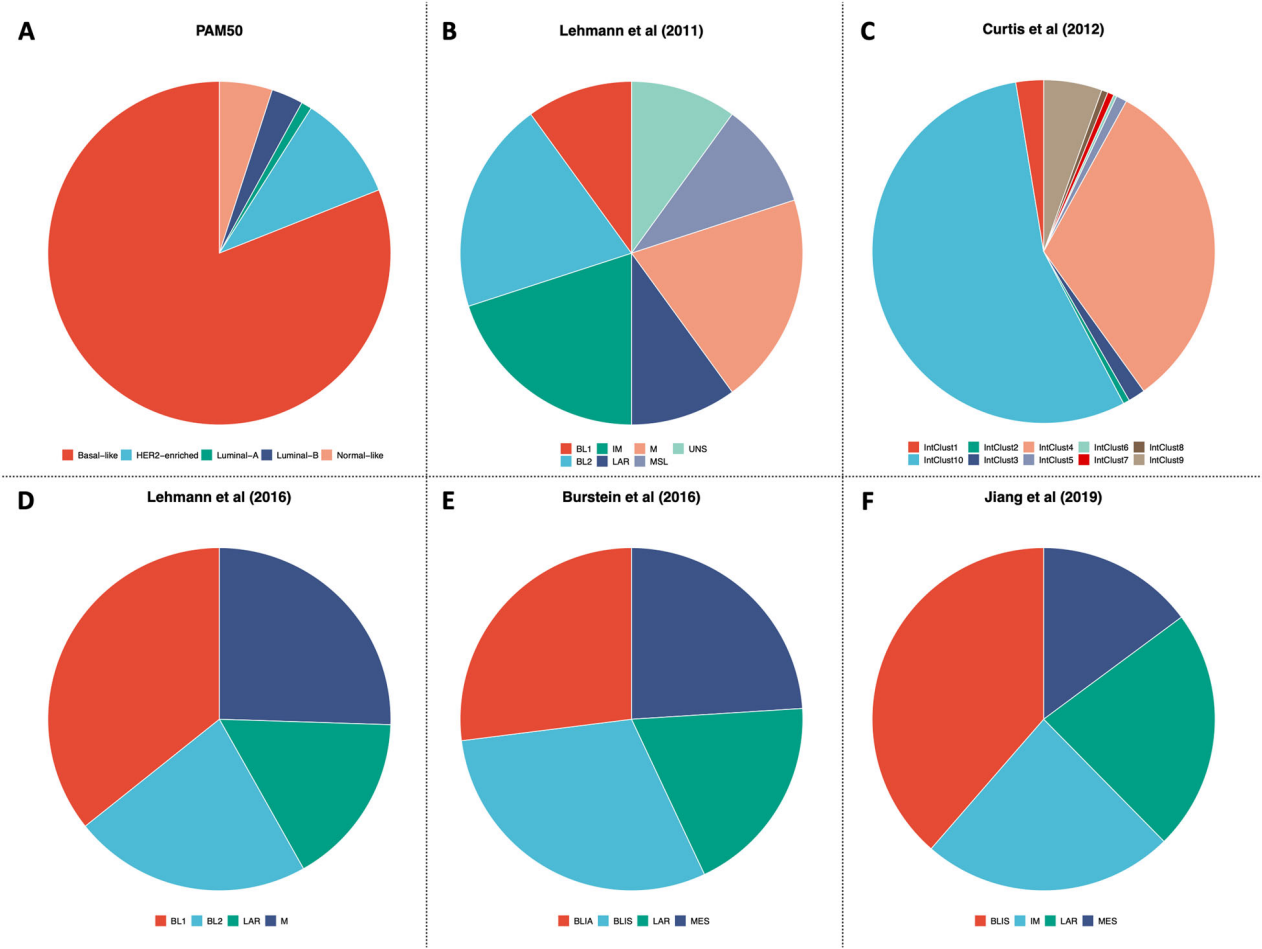
Triple-negative breast cancer molecular subtypes across studies. Transcriptomic-based and gene expression-based subtypes of triple-negative breast cancer (TNBC) according to PAM50^28^ (**a**) and defined by Lehmann et al.^20,22^ (**b** and **d**), Curtis et al.^24^ (**c**), Burstein et al.^23^ (**e**), and Jiang et al.^25^ (**f**). BLIA basal-like immune-activated, BLIS basal-like immunosuppressed, BL1 basal-like 1, BL2 basal-like 2, IM immunomodulatory, IntClust integrative clusters, LAR luminal androgen receptor, M mesenchymal, MES mesenchymal, MSL mesenchymal stem-like, UNS unstable. Figures was generated by reanalysis of publicly available studies and open-source platforms (cBioPortal: https://www.cbioportal.org).

Besides the histopathological differences, TNBC displays great heterogeneity also at transcriptomic level. The landmark study by Lehmann et al. ^20^ identified seven clusters of TNBC, namely basal-like 1 (BL1), basal-like 2 (BL2), immunomodulatory (IM), mesenchymal (M), mesenchymal stem-like (MSL), luminal androgen receptor (LAR), and unstable (UNS). Among the basal-like subtypes, BL1 is enriched in cell cycle regulator and DNA damage response pathways, while the BL2 shows high levels of growth factor and metabolic pathways, as well as an increased myoepithelial marker expression. The IM subtype is characterized by immune cell processes and immune signaling cascades. Although the M and MSL subtypes are quite similar at transcriptomic level being enriched for genes implicated in cell motility and EMT, the MSL subtype shows lower expression of genes associated with cellular proliferation and is enriched for genes related to mesenchymal stem cells. Lastly, the LAR subtype has luminal-like gene expression pattern, despite ER negativity. Albeit Lehmann et al. ^20^ had provided a proof-of-concept for personalized therapies in TNBC, further studies did not confirm a prognostic value for these subtypes^21^. Accordingly, subsequent efforts^21–25^ refined the TNBC molecular clusters into four tumor-specific subtypes (Fig. 1), each presenting different GEP, response to standard treatments and prognosis. These achievements were mainly gained thanks to the application of multi-omics profiling and single-cell analysis technologies, which allow preventing samples’ contamination by tumor-infiltrating lymphocytes (TILs) and other tumor microenvironment (TME) components^26,27^.

Along with transcriptional heterogeneity, TNBC is also characterized by complex genomes, dictated by high genetic instability and intricate patterns of copy number alterations and chromosomal rearrangements^28–32^. TNBCs present few highly recurrently mutated genes, being enriched in somatic mutations of tumor suppressor genes such as *TP53* and phosphatase and tensin homolog (*PTEN*). Conversely, driver alterations in genes of the phosphoinositide-3-kinase (PI3K)/AKT pathway, including *PIK3CA* mutations, have been described in ∼10% of cases^28^. Moreover, genomic analysis of the TNBC residual disease specimens after neoadjuvant chemotherapy (NAC) revealed at least one potentially-targetable genetic alteration^33^. Lately, Bareche et al. ^34^ reported the genomic alterations characteristic of each TNBC molecular subtype. BL1 tumors have high levels of chromosomal instability, high rate of *TP53* mutations (92%), copy-number gains and amplifications of *PI3KCA* and *AKT2*, and deletions in genes involved in DNA repair mechanisms. Conversely, the LAR subtype is characterized by higher mutational burden and enrichment in mutations of *PI3KCA*, *AKT1* and *CDH1* genes. Mesenchymal and MSL subtypes are associated with higher signature score for angiogenesis. As expected, the IM group showed high expression levels of immune response-associated signatures and checkpoint inhibitor genes, including cytotoxic T-lymphocyte-associated antigen-4 (CTLA-4), programmed cell death protein-1 (PD-1), and PD-ligand 1 (PD-L1). Such enrichment in the IM tumors should be related to the contamination by the immune infiltrate^22^. Of note, LAR subtype was associated with a worst prognosis, while IM subtype showed to be associated with a better prognosis^34^. In addition, it is relevant to underline that when Bareche et al. tried to reproduce Lehmann’s TNBC classification, BL1, IM, LAR, M, and MSL were the more stable subtypes. On the other hand, BL2 and UNS subtypes lacked reproducibility, as already observed in previous studies^21,35^.

Considering the great genomic complexity and heterogeneity^28–31^, analysis of a single genetic alteration might not be informative of the mutational processes driving TNBC. Accordingly, the application of mathematical models and computational frameworks allowed deciphering and identifying mutational signatures^36–38^. By analyzing the patterns of single nucleotide variants, pivotal studies led to the identification of two mutational signatures that were consistent with the activity of the apolipoprotein B mRNA editing enzyme catalytic polypeptide-like (APOBEC) family of deaminases. APOBEC enzymes activity has a central role in tumorigenesis, leading to subclonal expansion and intratumor heterogeneity across several tumors^39^. In BC, the role of APOBEC-associated mutagenesis has been extensively studied in ER-positive disease^40^, while scant information on TNBC is available. Therefore, additional research is needed to fully elucidate prognostic and therapeutic implications of mutational signatures in TNBC.

Lastly, histopathological and genomic characterization of tumor biopsy specimens can present several limitations, including a limited representativeness of the entire tumor mutational repertoire and its heterogeneity, technical issues in tissue processing and mutation detection, and low feasibility in some clinical circumstances^41^. Accordingly, some tools, referred to as “liquid biopsies”, have been implemented to identify and quantify tumor fractions released into the peripheral blood, including circulating tumor cells (CTCs), exosomes, and circulating tumor DNA (ctDNA)^42^. Different studies highlighted that CTCs-based and ctDNA-based liquid biopsies are able to real-time monitor disease evolution and identify patient with high-risk of disease recurrence and poor prognosis^43–45^. In TNBC, ctDNA-based genome-wide profiling demonstrated to be useful in characterizing tumor-specific alterations, as well as in predicting patient prognosis^46–48^. Considering that these observations mainly derive from retrospective and secondary analyses, further prospective trials investigating new treatments matched with liquid biopsy-assessed genomic alterations are warranted.

### Biomarkers-driven treatments in TNBC

**Fig. 2:**
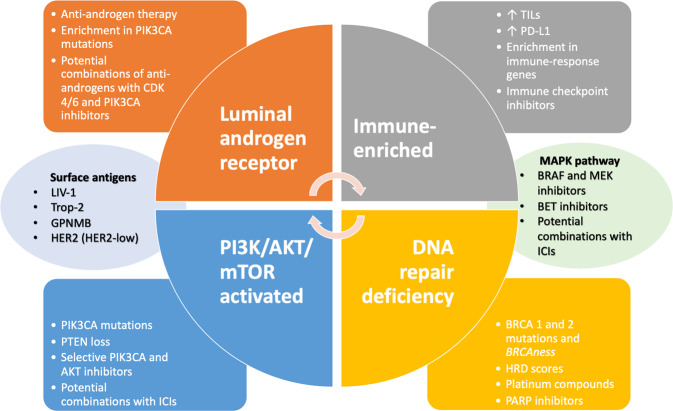
Biomarker-driven therapeutic approaches in triple-negative breast cancer.

The history of TNBC has observed several attempts to identify biomarkers capable to refine the patients’ selection and predict responses to standard and innovative therapies. Discovery and clinical implementation of new drugs in biomarker-driven manner is essential to decrypt the multitude mechanisms of resistance conferring poorer prognosis to TNBC patients (Fig. 2).

#### Tackling hormone receptors in TNBC: the androgen pathway

Pivotal studies on TNBC led to the identification of the LAR subtype^20–22^. Representing 10–15% of all TNBCs, androgen receptor (AR)-positive tumors are characterized by low proliferation rates and luminal-like gene expression profile, being inherently resistant to chemotherapy^20–23,34,49^. By contrast, AR expression assessed by IHC does not seem to imply a worse prognosis^50^. AR is a ligand-activated transcription factor that exerts genomic and non-genomic effects on cells by involving different intracellular signaling pathways and promoting tumor proliferation and invasiveness^51,52^.

AR has been identified as an appealing target for the TNBC treatment, prompting the clinical implementation of anti-androgens molecules. Androgen manipulation can be generally obtained with the use of direct AR-blockers. The non-steroidal AR inhibitor bicalutamide was tested in a phase II trial^53^ that included patients with pretreated, metastatic AR-positive TNBC, using a minimal threshold of IHC expression of 10%. The study failed to show any benefit, corresponding to a clinical benefit rate (CBR) of 18% and median progression-free survival (mPFS) of 12 weeks. Based on the possibility to overcome acquired androgen-resistance across cytoplasmatic and nuclear AR-related pathways, the non-steroidal anti-androgen enzalutamide was tested in a phase II trial in patients with pretreated TNBCs with nuclear AR staining >0%^54^. In the overall population, CBR and mPFS was 25% and 2.9 months, respectively. Interestingly, TNBCs expressing AR more than 10% and enriched for an androgen-driven gene signature seemed to be more sensitive to enzalutamide (mPFS 32 vs. 9 weeks)^55^. The clinical experience with the steroidal inhibitor of androgenesis abiraterone appeared consistent with enzalutamide, for AR of 10% or more, resulting in a mPFS of 2.8 months and CBR of 20%^56^.

Taken together, these data suggest a narrow benefit of androgen blockers in TNBC. Although androgen blockade has demonstrated a potential value in AR-positive TNBC, the predictive role of AR expression alone needs a better characterization. Accordingly, a deeper androgen suppression or AR degradation could improve the anti-tumoral activity, as being tested in ongoing clinical trials (Table 1). Moreover, co-targeting possible mechanisms of escape in alternative pathways implicated in androgen-resistance can represent an attractive strategy, as validated in ER-positive BC with cyclin-dependent kinase (CDK) 4/6 inhibitors and PI3K blockers^57,58^. Considering that the LAR subtype demonstrated highly sensitivity to CDK 4/6 inhibition in preclinical models^59^, as well as higher mutational burden and enrichment in mutations of the PI3K pathway^34,60,61^, clinical trials testing CDK 4/6 and PI3K-selective inhibitors in combination with novel anti-androgens are currently ongoing (Table 1).

**Table 1:** Selected ongoing phase II or III clinical trials in TNBC.

Agent(s)	Target(s)/pathway(s)	Phase	Setting	Sample size	Estimated study completion	ClinicalTrials.gov Identifier
Immunotherapy						
Pembrolizumab IL-12 gene therapy L-NMMA Chemotherapy^#^	PD-1 IL-12	II	(Neo)-adjuvant	43	Aug 2020	NCT04095689
HLX10 Chemotherapy^##^	PD-1	III	(Neo)-adjuvant	522	Apr 2027	NCT04301739
Atezolizumab Ipatasertib Paclitaxel	PD-L1 AKT	III	Advanced/metastatic	1155	Oct 2025	NCT04177108
Spartalizumab LAG525 Carboplatin	PD-1 LAG-3	II	Advanced/metastatic	88	Jan 2021	NCT03499899
Toripalimab Nab- paclitaxel	PD-1	III	Advanced/metastatic	660	Feb 2022	NCT04085276
Camrelizumab Famitinib Carboplatin	PD-1	II	Advanced/metastatic	46	Jan 2021	NCT04129996 (FUTURE-C-PLUS)
Lacnotuzumab Gemcitabine Carboplatin	M-CSF	II	Advanced/metastatic	50	Mar 2020	NCT02435680
Nivolumab Capecitabine	PD-1	II	Post-neoadjuvant without pCR	45	Dec 2022	NCT03487666 (OXEL)
Pembrolizumab Imprime PGG	PD-1 Dectin	II	Advanced/metastatic	64	Nov 2021	NCT02981303
Avelumab	PD-L1	III	Adjuvant	335	June 2023	NCT02926196 (A-BRAVE)
Pembrolizumab Tavokinogene telseplasmid (intratumoral)	PD-1	II	Advanced/metastatic	25	Jan 2020	NCT03567720 (KEYNOTE-890)
Nivolumab Ipilimmumab Capecitabine Radiation therapy	PD-1 CTLA-4	II	Adjuvant	98	March 2022	NCT03818685 (BreastImmune03)
Durvalumab CFI-400945	Pd-L1 Plk4	II	Advanced/metastatic	28	Dec 2022	NCT04176848
KN046 Nab-paclitaxel	PD-L1 CTLA-4	I/II	Advanced/metastatic	90	Sept 2021	NCT03872791
Atezolizumab Ipatasertib Ladiratuzumab-Vedotin Bevacizumab Cobimetinib RO6874281 Selicrelumab Chemotherapy	PD-L1 AKT LIV-1 VEGF MEK IL-2 CD40	I/II	Advanced/metastatic	310	Aug 2021	NCT03424005** (MORPHEUS-TNBC)
PF-04518600 Avelumab Binimetinib Utomilumab	OX-40 PD-L1 MEK 4-1BB/CD137	II	Advanced/metastatic	150	June 2023	NCT03971409 (inCITe)
Atezolizumab Cobimetinib Nab-paclitaxel/paclitaxel	PD-L1 MEK	II	Advanced/metastatic	269	Apr 2020	NCT02322814
Durvalumab Oleclumab Paclitaxel Carboplatin	PD-L1 CD73	I/II	Advanced/metastatic	171	Dec 2022	NCT03616886 (SYNERGY)
CAN04 Chemotherapy	IL1RAP	I/II	Advanced/metastatic	100	Oct 2020	NCT03267316 (CANFOUR)
Sarilumab Capecitabine	IL-6	I/II	Advanced/metastatic	50	June 2020	NCT04333706 (EMPOWER)
NKTR-214 Nivolumab Ipilimumab	CD122 PD-1 CTLA-4	I/II	Advanced/metastatic	780	Dec 2021	NCT02983045 (PIVOT 02)
Nivolumab Ipilimumab	PD-1 CTLA-4	II	Advanced/metastatic	30	Oct 2022	NCT03789110 (NIMBUS)
PARP inhibitors and other DNA modulating agents						
Niraparib Pembrolizumab	PARP PD-1	I/II	Advanced/metastatic	121	Mar 2020	NCT02657889 (TOPACIO)
Olaparib	PARP	III	Adjuvant	1836	Nov 2020	NCT02032823 (OlympiA)
Olaparib AZD6738 AZD1775	PARP ATR WEE1	II	Advanced/metastatic	450	Nov 2020	NCT03330847
Olaparib Durvalumab	PARP PD-L1	II	Advanced/metastatic	28	Dec 2020	NCT03801369
Olaparib Durvalumab Bevacizumab	PARP PD-L1 VEGF	I/II	Advanced/metastatic gBRCAm	427	Sep 2022	NCT02734004 (MEDIOLA)
Talazoparib Avelumab	PARP PD-L1	II	Advanced/metastatic	242	Aug 2020	NCT03330405
Olaparib Durvalumab	PARP PD-L1	II	Advanced/metastatic	60	Apr 2020	NCT03167619 (DORA)
Olaparib Platinum-based CT	PARP	II/III	(Neo)-adjuvant	527	Jan 2032	NCT03150576 (PARTNER)
Olaparib Durvalumab AZD6738	PARP PD-L1 ATR	II	(Neo)-adjuvant	81	Dec 2025	NCT03740893 (PHOENIX)
Olaparib	PARP	II	Advanced/metastatic	91	Dec 2020	NCT00679783
Olaparib Durvalumab	PARP PD-L1	I/II	(Neo)-adjuvant	25	Apr 2020	NCT03594396
Talazoparib ZEN003694	PARP Bromodomain	II	Advanced/metastatic	29	Jan 2021	NCT03901469
Talazoparib	PARP	II	Advanced/metastatic	40	Aug 2021	NCT02401347
Veliparib Cisplatin	PARP	II	Advanced metastatic	333	Oct 2021	NCT02595905
Pembrolizumab Olaparib Gemcitabine Carboplatin	PD-1 PARP	II/III	Advanced/metastatic	932	Jan 2026	NCT04191135
Olaparib	PARP	II	Advanced/metastatic	39	Nov 2021	NCT03367689
PI3K/mTOR/AKT/PTEN pathway						
Tak-228 Tak-117 Cisplatin Nab-paclitaxel	TORC 1/2 PI3Kα	II	Advanced/metastatic	20	June 2022	NCT03193853
LY3023414 Prexasertib	PI3K/mTOR CHEK1	II	Advanced/metastatic	10	Aug 2021	NCT04032080 (ExIST)
Everolimus Carboplatin	mTOR	II	Advanced/metastatic	72	June 2021	NCT02531932
Ipatasertib Paclitaxel	AKT	II/III	Advanced/metastatic	450	Dec 2021	NCT03337724 (IPATunity130)
Alpelisib Nab-paclitaxel	PIK3CA	II	Advanced/metastatic	62	Dec 2021	NCT04216472
Capivasertib Paclitaxel	AKT	III	Advanced/metastatic	800	Sept 2021	NCT03997123 (CapItello290)
IPI-549 Atezolizumab Bevacizumab Nab-paclitaxel	PI3K-gamma PD-L1 VEGF	II	Advanced/metastatic	90	Aug 2022	NCT03961698 (MARIO-3)
Gedatolisib Talazoparib	PI3K/mTOR PARP	II	Advanced/metastatic	54	May 2022	NCT03911973
Vistusertib Selumetinib	mTORC1/2 MEK	II	Advanced/metastatic	118	Mar 2020	NCT02583542 (TORCMEK)
Capivasertib Ceralasertib Adavosertib Olaparib	AKT ATR WEE1 PARP	II	Advanced/metastatic	64	Mar 2020	NCT02576444 (OLAPCO)
RAS/MAPK/ERK						
ONC 201	ERK AKT	II	Advanced/metastatic	90	Dec 2027	NCT03394027
Antibody-drug conjugates						
Sacituzumab govitecan Chemotherapy	Trop2	III	Advanced/metastatic	529	July 2020	NCT02574455 (ASCENT)
CAB-ROR2-ADC BA3021	ROR2	I/II	Advanced/metastatic	120	May 2022	NCT03504488
SKB264	Trop2	I/II	Advanced/metastatic	78	Dec 2022	NCT04152499 (A264)
EnfortuMab Vedotin	Nectin-4	II	Advanced/metastatic	240	Apr 2023	NCT04225117 (EV-202)
Androgen pathway						
Orteronel	17α-hydroxylase	II	Advanced/metastatic	71	Feb 2020	NCT01990209
Enobosarm Pembrolizumab	AR PD-1	II	Advanced/metastatic	29	Nov 2020	NCT02971761
Bicalutamide Palbociclib	AR PARP	II	Advanced/metastatic	51	Nov 2020	NCT02605486
Enzalutamide Taselisib	AR PI3K	I/II	Advanced/metastatic	73	Dec 2021	NCT02457910
Enzalutamide Alpelisib	AR PIK3CA	II	Advanced/metastatic	28	Dec 2020	NCT03207529
Bicalutamide	AR	II	Advanced/metastatic	262	Dec 2020	NCT03055312 (SYSUCC-007)
Enzalutamide	AR	II	Adjuvant	50	May 2020	NCT02750358
Enzalutamide Paclitaxel	AR	II	Neoadjuvant	37	Sept 2021	NCT02689427
Bicalutamide Ribociclib	AR PARP	I/II	Advanced/metastatic	11	Sept 2021	NCT03090165
Darolutamide Capecitabine	AR	II	Advanced/metastatic	90	Sept 2021	NCT03383679 (START)
Orteronel	17α-hydroxylase	II	Advanced/metastatic	71	Feb 2020	NCT01990209

IL-2 gene therapy refers to Adenoviral-mediated IL-12. L-NMMA, NG-monomethyl-L-arginine, inhibitor of the nitric oxide synthetase. Famitinib, inhibitor of multiple tyrosine kinase receptor anti c-Kit, vascular endothelial growth factor receptor 2 and 3, platelet-derived growth factor receptor and FMS-like tyrosine kinases Flt1 and Flt3. M-CSF, macrophage colony-stimulating factor. pCR, pathological complete response. Tavokinogene telseplasmid is aDNA plasmid that encodes genes for both the p35 and p40 subunits of the heterodimeric human interleukin 12 (hIL-12) protein.

*PLK4* Polo-like kinase 4, *IL1RAP* Interleukin 1 Receptor Accessory Protein, *pCR* pathological complete response, *DDR* DNA damage repair.

^#^standard neoadjuvant regimen with anthracyclines and taxanes.

^##^nab-paclitaxel, carboplatin, doxorubicin/epirubicin and cyclophosphamide.

**umbrella study evaluating the efficacy and safety of multiple immunotherapy-based treatment combinations.

#### Refining the selection per biomarker in targeting PI3K-AKT-mTOR pathway

The PI3K-AKT-mTOR (PAM) pathway is frequently dysregulated in cancer, promoting cell proliferation and tumorigenesis. The activation of the PAM pathway can result from the oncogenic activation of growth factor receptors and direct oncogenic activation of the PAM proteins or their regulators, including PTEN and inositol polyphosphate 4-phosphatase (INPP4B)^62^.

PIK3CA is one of the most frequent mutated genes in TNBC (∼10%), being enriched in basal-like and LAR subtypes^24,25,28–34,36,63^. Notably, metastatic TNBCs carrying PIK3CA mutations seem to have better overall survival (OS) than wild-type counterparts. However, such observation could be partially explained by an enrichment in PIK3CA mutations in luminal BCs that loss ER expression in the metastatic setting^63^. Furthermore, loss-of-function of PTEN and INPP4B have been described in up to one-third of the TNBCs, especially in BLBC where heterozygous loss of PTEN has been identified in >45% of cases^28^.

Despite the key role in tumorigenesis, the clinical implementation of drugs targeting PAM molecules has resulted in disappointing results so far. The perception is that the regulation of single downstream effectors may activate uncontrolled resistance feedback loops. Conversely, the combination of multiple agents against PAM molecules has often resulted in unacceptable toxicity, mainly with mTOR and pan-PI3K inhibitors^64,65^. Thus, biomarker selection and more selective inhibitors were argued. In a phase I/II trial of patients with HER2-negative BC^66^, the α-selective PI3K inhibitor alpelisib combined with nab-paclitaxel provided the largest benefit in the population harboring PIK3CA mutations (mPFS 13 months). Similar results were obtained in the phase II randomized trial LOTUS, where the AKT inhibitor ipatasertib combined with paclitaxel provided meaningful benefit in patients carrying PIK3CA/AKT1/PTEN alterations^67,68^. Similarly, the phase II randomized study PAKT confirmed an improvement in PFS and OS in the biomarker-enriched (PIK3CA/AKT1/PTEN) population treated with the AKT inhibitor capivasertib added to first-line chemotherapy^69^.

As dominated by PAM alterations, a further step to target TNBC per biomarker will be driven by the deeper understanding of the collateral activated pathways and biological implications of PAM dysregulations and pharmacological inhibition. In this perspective, combined approaches with AR, CDK 4/6, and double PI3K/mTOR inhibitors are under investigation (Table 1). Furthermore, considering the metabolic function of PAM signaling in the insulin response, it has been proposed that the reactivation of the insulin feedback induced by PI3K inhibitors may reactivate the PI3K-mTOR signaling axis in tumors, thereby compromising treatment effectiveness^70^. Accordingly, a medical intervention capable to reduce the insulin secretion could enhance the benefit of PI3K inhibitors, for instance through a switch of the metabolic use of nutrients on a ketogenic profile^71^. Despite preliminary, these findings support a possible synergistic action of PAM agents and dietary interventions, as investigated in several clinical trials^72^.

#### Mitogen-activated protein kinase pathway

Mitogen-activated protein kinase (MAPK) cascades is a finely-controlled signal transduction pathway consisting of phosphoserine/threonine kinases that mediates the cellular response to external signals^73^. The pathway proceeds from the sequential phosphorylation of several molecules (Erk, Mek, and Raf), and is finely regulated by GTPase proteins, including the RAS proteins.

Alterations in genes encoding for components of the MAPK pathway, including KRAS, BRAF, and MEK1/2, are described in less than 2% of TNBCs^28^. However, somatic alterations of regulatory proteins that contribute to the oncogenic dysregulation of MAPK pathway have been more commonly reported, such as the negative regulator of ERK1/2 and JNK1/2 dual specificity protein phosphatase 4 (DUSP4)^33,74^. In TNBC, the regulation of MAPK has demonstrated to be potentially targetable, suppressing redundant pathways converging on the cascade. For instance, EGFR overexpression was shown to upregulate the Ras/MAPK signaling, becoming an appealing therapeutic target^75^. However, neither monoclonal antibodies (mAbs) nor tyrosine kinase inhibitors (TKIs) targeting EGFR demonstrated meaningful activity in TNBC in phase II/III trials^76–81^. Some possible explanations can be offered to explain the clinical failure of anti-EGFR agents in TNBC^82^. First, the EGFR signaling may change during disease progression, with low EGFR expression in the metastatic cells, despite an overexpression in primary tumor^83^. Second, the significant interaction between EGFR and other oncogenic signaling pathways (MET, PI3K/mTOR, and MEK pathways) might render TNBC cells intrinsically resistant to EGFR-specific inhibition. In this way, further studies investigating new TKIs^84^, as well as novel combinatorial strategies are underway.

Moreover, given the central role of MAPK dysregulation in BC tumorigenesis, the opportunity to target MEK in TNBC patients with the selective inhibitor cobimetinib was explored in the phase II trial COLET. Again, the addition of cobimetinib to first-line paclitaxel did not demonstrate significant improvement in mPFS^85^. Interestingly, the biomarker explorative analysis showed a potential immunomodulatory effect of cobimetinib in increasing the immune infiltration within TME^86^. Consistently, the part 2 of COLET study was designed to test the combination of cobimetinib, nab-paclitaxel and atezolizumab, with preliminary signs of activity in the PD-L1-positive population^87^. Recently, the concomitant inhibition of MEK and bromodomain and extraterminal motif (BET) has shown synergistic activity in TNBC preclinical models overexpressing the neuroendocrine-associated oncogene MYCN^88^, providing a rationale to explore this combination in patients with MYCN-positive TNBC.

Comparable to other MAPK pathway components, somatic mutations of BRAF have been reported in less than 1% of BCs^89^. Although no specific data on TNBC patients are available, compelling evidence suggests potential clinical benefit for BRAF inhibition in tumors carrying V600E BRAF mutations^90,91^. Overall, the evidence converges on the concept that the pharmacological manipulation of MAPK in TNBC can be effective when the MAPK pathway is dysregulated, commonly for genomic alterations of its regulators. In this way, ongoing trials are exploring the opportunity to target MAPK with single agents in biomarker-selected patients for MAPK dysregulations, such as DUSP4 expression^92^, as well as the potential immune-enhancing effect by the pharmacological inhibition of Ras/MAPK pathway^93^ (Table 1).

#### Tailoring the homologous recombination repair mechanisms and its regulators: the BRCAness paradigm

Defects in double-stranded DNA (dsDNA) repair mechanisms are characteristic of TNBC, as a result of either germline or somatic mutations in BRCA1/2 and other genes involved in DNA repair^94^. Germline mutations in BRCA1/2 occur in ∼10% of TBNC patients and increase the lifetime risk of BC to 60–70%^95–97^. Notably, BRCA1-mutated BCs usually display a basal-like phenotype^98^. Key feature of BRCA1/2 mutant TNBC is the deficiency of homologous recombination repair (HRR), making essential other DNA repair machineries to maintain the integrity of the genome. Similar to BRCA1/2, HR deficiency (HRD) can result from the loss of several proteins, contributing to the acquisition of a BRCA-like phenotype (also defined *BRCAness*)^99^. The term has been introduced to define the situation in which an HRR defect exists in a tumor in the absence of a germline BRCA1/2 mutation, conferring sensitivity to poly ADP-ribose polymerase (PARP) inhibitors (PARPis) based on the principle of synthetic lethality^99–101^. Computational modeling of sequencing data allowed to identify additional tumors with somatic loss or functional deficiency of BRCA1/2 where no mutations were detected, potentially expanding the BC population amenable to PARPis^102^.

Nowadays, germline pathogenetic mutations of BRCA1/2 are the only clinically validated biomarkers of sensitivity to PARPis, based on the results of clinical trials testing olaparib, talazoparib and veliparib in metastatic BC (Table 2)^103–106^. In addition, patients carrying BRCA pathogenic mutations showed to derive a greater benefit with DNA-targeting cytotoxic agents, as demonstrated in the phase III trial TNT that compared docetaxel to carboplatin in the first-line setting of metastatic TNBC^107^. Pre-specified analysis of biomarker-treatment interaction assessed the predictive role of germinal BRCA mutations and *BRCAness* alterations, including somatic BRCA1 methylation and HRD mutational signature (per Myriad assay)^107^. If on one hand germinal BRCA mutational status was able to predict the responses to carboplatin with a doubled overall response rate (ORR), on the other no difference could be detected by utilizing the other proposed biomarkers. Notably, patients with non-basal-like tumors (according to PAM50) derived greater benefit with docetaxel. Apparently, the benefit observed with platinum compounds seems applicable to other DNA-targeting agents, including anthracyclines, as showed in the INFORM trial that compared doxorubicin and cisplatin in the neoadjuvant setting^108^. In this trial, the single-agent platinum compound did not improve pCR rates compared to doxorubicin plus cyclophosphamide. When head-to-head comparisons have been performed across different agents, data suggest that the selection for HRD can predict equally a benefit to DNA-disrupting agents, regardless of their pharmacological class. The phase II trial GeparOLA assessed the efficacy of neoadjuvant paclitaxel and olaparib vs. paclitaxel and carboplatin in patients with HRD: overall, the study failed to show a difference in terms of pCR^109^. Eventually, the combination of PARPis and carboplatin did not result in a synergistic activity, across different settings of care, providing none or narrow added clinical benefit, at the cost of increased toxicity^110,111^.

**Table 2:** Main results of phase II/III trials testing PARP inhibitors in breast cancer.

Drug(s)	Phase	*N*	Population enrolled	Design	Primary endpoint	Results	Trial
Olaparib	III	302	Advanced gBRCA, HER2 negative, ≤ 2 prior lines of CT	Olaparib vs TPC	PFS	Median PFS (mo) 7.0 vs 4.2 Median OS (mo) 19.3 vs 17.1 ORR 59.9% vs 28.8%	OlympiAD (NCT02000622)
Olaparib	II	102	Neoadjuvant therapy for HER2 negative BC with gBRCA or tBRCA and/or high HRD score	Olaparib + paclitaxel → AC vs Carboplatin+ paclitaxel → AC	pCR	pCR 55.1% vs 48.6%	GeparOLA (NCT02789332)
Veliparib	III	634	Neoadjuvant therapy for stage II/III TNBC	Carboplatin + paclitaxel + veliparib → AC vs carboplatin+ paclitaxel + placebo →AC vs placebo+placebo + paclitaxel→ AC	pCR	pCR 58% vs 53% vs 31%	BrighTNess (NCT02032277)
Veliparib	II	116	Neoadjuvant therapy for stage II/III TNBC	Carboplatin + paclitaxel + veliparib/placebo → AC	pCR	pCR 51% vs 26%	I-SPY 2 (NCT01042379)
Veliparib	II	290	Advanced gBRCA 0–2 prior lines of CT	Carboplatin+ paclitaxel + veliparib vs carboplatin+ paclitaxel + placebo vs temozolamide + veliparib	PFS	Median PFS (mo) 14.1 vs 12.3 vs 7.4 Median OS (mo) 28.3 vs 25.9 vs 19.1 ORR 77.8% vs 61.3% vs 28.6%	BROCADE (NCT01506609)
Veliparib	III	513	Advanced gBRCA, HER2 negative 0–2 prior lines of CT	Carboplatin + paclitaxel + veliparib vs carboplatin + paclitaxel + placebo	PFS	Median PFS (mo) 14.5 vs 12.6 Median OS (mo) 33.5 vs 28.2 ORR 75.% vs 74.1%	BROCADE3 (NCT02163694)
Talazoparib	III	431	Advanced gBRCA, HER2 negative ≤ 3 prior lines of CT	Talazoparib vs TPC	PFS	Median PFS (mo) 8.6 vs 5.8 Median OS (mo) 22.3 vs 19.5 Response rate 62.6% vs. 27.2%	EMBRACA (NCT01945775)
Niraparib	III		Advanced gBRCA, HER2 negative ≤ 2 prior lines of CT	Niraparib vs TPC	PFS	Ongoing (no results available)	BRAVO (NCT01905592)

*AC* doxorubicin + cyclophosphamide, *CT*, chemotherapy, *gBRCA* germline BRCA mutation, *HRD score* Homologous Recombinant Deficiency score, *iDFS* invasive disease free survival, *mo* months, *ORR* objective response rate, *pCR* pathological complete response, *PFS* progression-free survival, *OS* overall survival, *tBRCA* somatic BRCA mutation, *TPC* treatment of physician’s choice chemotherapy.

The expansion of the concept of *BRCAness* beyond BRCA means to deepen the identification and study of key regulatory mechanisms of HRR, in order to develop predictive biomarkers for DNA disrupting agents and discover new pharmacological targets. ATR and its downstream effector (e.g., CHK1, WEE1, Aurora A, Polo-Like Kinase 1) have been proposed as possible modulators of *BRCAness*, potentially extending the spectrum of DNA-targeting and sensitivity to PARPis to a greater proportion of BC patients. These effectors seem to link the cell cycle control and the DNA damage response, both commonly disrupted mechanisms underlying tumorigenesis. Preliminary results have been reported for the Aurora A kinase selective inhibitor alisertib combined with paclitaxel^112^ and the Aurora A inhibitor ENMD-2076^113^ in phase I trials. Similarly, a combination trial of TNBC, with a pre-specified enrichment of BRCA mutated patients, assessed the benefit of the ATR-blocker M6620 with cisplatin, showing preliminary encouraging results^114^. The only suggested biomarker emerged for the HRR modulator targeting CHK1, GDC-0425, is TP53-mutated, tested as a chemotherapy-potentiating agent in patients with solid tumors, including TNBC^115^.

So far, no clinical experience with multiple HRR modulators is available, and clinical trials are ongoing. For the potential role in predicting a benefit with chemotherapy and PARPis, HRD has been proposed for the biomarker-enhanced design of clinical trials, with single agent PARPis and other modulators of the DNA damage response as single agents or in combination (Table 1). In addition, the preclinical evidence of a potential role for PI3K blockade in determining *BRCAness* phenotype in BRCA1/2-proficient TNBC^116^ would potentially expand the BC population likely benefiting from PARPis. However, a phase I trial testing the combination of buparlisib and olaparib reported modest results in terms of efficacy with relevant additional toxicity^117^. Lastly, preclinical evidence showed a potential immunomodulant activity for PARPis, including PD-L1 upregulation on cancer cell and activation of immune-response pathways such as STING^118–120^, leading to the design of clinical trials testing the combination of PARPis and immune checkpoint inhibitors (ICIs)^121,122^. Other agents targeting DNA repair proteins in combination with immunotherapy are also being currently investigated (Table 1).

#### Toward a precision immunotherapy for TNBC

The clinical landscape of drug development for immunotherapy agents in TNBC is complex and wide. Considering the key role of immune system in influencing responses to standard chemotherapy and prognosis of TNBC^123–126^, several trials tested ICIs targeting PD-1/PD-L1, showing limited activity as monotherapy and promising results in combination with chemotherapy (Table 3)^127–134^. The phase III trial IMpassion130, which tested the combination of atezolizumab with nab-paclitaxel in the first-line metastatic setting, established a potential role of immunotherapy in patients with PD-L1-positive TNBC, defined as ≥1% in tumor-infiltrating immune cells based on the IHC SP142 assay^135,136^.

**Table 3:** Main results of clinical trials testing immune checkpoint inhibitors alone or in combination with chemotherapy in advanced/metastatic triple-negative breast cancer.

Drug(s)	Phase	*N*	PD-L1 stratified	ORR (%)	Median PFS	Median OS	Trial
Monotherapy							
Pembrolizumab	I	32	≥1% TC	18.5	1.9 (1.7–5.5)	11.2 (5.3-NR)	KEYNOTE-012 (NCT01848834)
			Overall	5.3	2.0 (1.9–2.0)	9.0 (7.6–11.2)	
Pembrolizumab	II	170	≥1 CPS	5.7	2.0 (1.9–2.1)	8.8 (7.1–11.2)	KEYNOTE-086-A (NCT02447003)
			Negative	4.7	1.9 (1.7–2.0)	9.7 (6.2–12.6)	
Pembrolizumab	II	84	≥1 CPS	21.4	2.1 (2.0–2.2)	18.0 (12.9–23.0)	KEYNOTE-086-B (NCT02447003)
			Overall	9.6	2.1 (1.33–1.92)	9.9 (0.82–1.15)	
Pembrolizumab	III	622	≥1 CPS	12.3	2.1 (1.08–1.68)	10.7 (0.69–1.06)	KEYNOTE-119 (NCT02555657)
			≥10 CPS	17.7	2.1 (0.82–1.59)	12.7 (0.57–1.06)	
			≥20 CPS	26.3	3.4 (0.49–1.18)	14.9 (0.38–0.88)	
			Overall	5.2	5.9 (5.7–6.9)	9.2 (4.3–NR)	
Avelumab	I	58	≥10% IC	22.2	NA	NA	JAVELIN (NCT01772004)
			<10% IC	2.6	NA	NA	
Atezolizumab	I	115	≥1% IC	10	1.4 (1.3–1.6)	8.9 (7.0–12.6)	NCT01375842
Combinations							
Pembrolizumab + eribulin	I/II	106	Overall	26.4	4.2 (4.1–5.6)	17.7 (13.7–NR)	ENHANCE-1 (NCT02513472)
			≥1 CPS (1 line)	34.5	6.1 (4.1–10.2)	21.0 (8.3–29.0)	
			<1 CPS (1 line)	16.1	3.5 (2.0–4–2)	15.2 (12.8–19.4)	
			≥1 CPS (2–3 line)	24.4	4.1 (2.1–4.8)	14.0 (11.0–19.4)	
			<1 CPS (2–3 line)	18.2	3.9 (2.3–6–3)	15.5 (12.4-18-7)	
Atezolizumab + nabpaclitaxel	I	33	Overall	39.4	9.1 (2.0–20.9)	14.7 (10.1–NR)	NCT01375842
Atezolizumab + nabpaclitaxel	III	902	Overall	56	7.2 (0.69–0.92)	21.0 (0.72–1.02)	IMpassion130 (NCT02425891)
			≥1% IC	58.9	7.5 (0.49–0.78)	25.0 (0.54–0.93)	

PFS and OS are expressed as median (95% confidence interval), in months.

*PFS* progression-free survival *OS* overall survival, *NR* not reached, *TC* tumor cells, *CPS* combined positive score, *IC* immune cells, *NA* not available.

So far, PD-L1 is the only biomarker applied in clinical practice for the selection of patients more likely to respond to anti-PD-1/PD-L1 ICIs. However, how to define the “PD-L1-positive” population in the clinical practice remains challenging. Even if the biomarker analysis of IMpassion130 showed a correlation between the expression of PD-L1 on tumor and immune-infiltrating cells, issues on the IHC assay to apply and the definition of the optimal threshold for “positivity” could still be arisen. When SP142 assay was compared with the other commercially available antibodies 22C3 (expressed as the percentage of viable staining positive to PD-L1 or tumor proportion score) and SP263 (PD-L1 staining on immune and tumor cells), the latter were both able to identify more patients with PD-L1-positive tumors^137^. Conversely, within the Impassion130 trial, the patients defined “PD-L1-positive” by 22C3 and SP263 clones derived lower PFS and OS benefit than with SP142. Furthermore, despite the clinical implementation of PD-L1 as a clinically useful biomarker, concerns have been raised on the broad utility of PD-L1 expression for selecting patients. As a dynamic biomarker, PD-L1 can be differentially expressed in primary and metastatic sites and responses are observed in PD-L1 negative patients as well^138^.

In the TNBC early setting, the role of PD-L1 becomes particularly controversial. In the phase III trial KEYNOTE-522, the addition of pembrolizumab to anthracycline, taxane and platinum neoadjuvant regimen improved pCR rates (64.8% vs. 51.2%)^139^, regardless of PD-L1 expression^140^. In another neoadjuvant phase III trial (NeoTRIPaPDL1), the addition of atezolizumab to carboplatin and nab-paclitaxel failed to show an improvement in pCR compared to standard chemotherapy alone^141^. Of note, the exploratory analysis identified the PD-L1 expression as the strongest predictor of response to the immune-chemotherapy combination. These contrasting results might be partially explained by the different chemotherapy regimens used. As highlighted in the TONIC trial^142^, the presence of an anthracycline or platinum compound as induction chemotherapy may create a more favorable TME and increase pCR to neoadjuvant PD-1 blockade in TNBC.

Overall, PD-L1 is still a suboptimal biomarker to properly select TNBC patients for immunotherapy-based treatments. Therefore, additional biomarkers have been proposed or are currently under investigation^143^.

Tumor mutational burden (TMB) has emerged as biomarker of improved survival in cancer patients receiving ICIs^144,145^. However, limited data are available on the value of TMB in BC, also considering the low proportion of hypermutated BCs^146^. A recent study on patients with metastatic TNBC treated with ICIs showed that high TMB (≥6 mutations/megabase) was significantly associated with longer PFS, but not OS^147^. Preclinical evidence suggested that neoantigen quality, rather than quantity, is the major determinant for inducing effective and durable immune responses and shaping response to ICIs^148^, as demonstrated in melanoma and lung cancer models^149^. Accordingly, further research is ongoing to develop pipelines for identifying specific mutational signatures that may be associated with anti-tumor immune response in BC^150–152^. As described above, an hypermutated phenotype in BC can be sustained with APOBEC-associated mutational processes in ∼60% of cases^36–39^, while only few hypermutated tumors present HRD (1%) or dysregulation of the DNA polymerase-epsilon (3.4%)^146^. This makes clear that HRD alone is not able to predict a hypermutated phenotype nor a pronounced likelihood of response to ICIs. Lastly, an in-silico analysis showed that immune infiltration was associated with TMB in tumors driven by recurrent mutations, but not in those driven by copy number alterations such as BCs^153^. Overall, further research is needed to clarify the role of these biomarkers in predicting ICIs efficacy in TNBC.

The spatial architecture and arrangement of TILs have been primarily addressed, initially assessing the presence, pattern and density and then moving to their functional characterization. While ICIs mainly act reinvigorating a pre-existing anti-tumor immune response, TILs density has been associated with ICIs activity in several solid tumors^154^. In BC, these findings were confirmed in patients with metastatic TNBC who were treated with pembrolizumab monotherapy in the phase II KEYNOTE-086 trial^155^, where TILs levels were independent predictors of response. In addition, the presence and density of pre-treatment and on-treatment stromal TILs were significantly associated with pCR in a cohort of the KEYNOTE-173 trial, which assessed the benefit of pembrolizumab added to NAC^156^. Similar results have been showed in exploratory biomarker analysis of the IMpassion130^157^ and KEYNOTE-119 trials^158^. Interestingly, the latter study showed that high TILs predicted favorable clinical outcomes in patients with metastatic TNBC treated with pembrolizumab, but not with chemotherapy, reinforcing the predictive value of this biomarker. Nevertheless, further evidence suggested that qualitative differences in TIL composition, as well as immune-related genetic signatures are able to fine-tune patients’ prognosis in TNBC and response to ICIs^154,159,160^. So far, no functional characterization of the immune infiltrate has been reported from controlled trials testing ICIs in TNBC.

#### Optimizing the delivery of cytotoxic agents via surface antigens: the model of antibody drug-conjugates

Drug delivery via antibody drug-conjugates (ADCs) sublimates the traditional concept of target in cancer treatment. So far, the most common use of mAbs in BC has been directed to an oncoprotein with a pathogenetic role in tumorigenesis. With the advent of ADCs, the identification of cell surface targets has now the explicit role to tag cancer cells, ensuring the univocal identification of the malignancy and providing a precise delivery of the conjugated payloads, either cytotoxic agents or biological molecules^161^. This demands a change in the perspective: identifying target molecules with a restricted expression on cancer cell surfaces, irrespective of their biological function. In the clinical setting, the identification and quantification of possible targets for ADCs have used IHC scores, both as a semiquantitative scale (e.g., 0 to 4+ for LIV-1 and Trop-2) or percentage of positive cells (e.g., GPNMB).

In BC, the pipeline of ADCs is being continuously growing^161^. Sacituzumab-govitecan-hziy is an ADC recognizing Trop-2 on cells to deliver the camptothecin-derived cytotoxic payload SN-38^162^. Trop-2 is a transmembrane calcium signal transducer and is overexpressed in many epithelial cancer, including nearly 90% of TNBCs^163,164^. The phase I/II trial IMMU-132-01 showed promising results in pretreated TNBC patients, with PFS and OS of 5.5 and 13 months, respectively^165^. On these bases, FDA grants accelerated approval to sacituzumab govitecan-hziy for metastatic TNBC treatment. Furthermore, the phase III trial ASCENT, which tested sacituzumab-govitecan-hziy against the investigators’ choice in third or further line, was recently stopped for the fulfillment of the study endpoints^166^. Another transmembrane glycoprotein, GPNMB, has been proposed as surface antigen for ADC, being enriched in ∼30% of TNBCs^167^. Glembatumumab-vedotin is an ADC (carrying the auristatin-derivate monomethyl auristatin E) directed toward GPNMB. In clinical studies, this ADC failed to improve the ORR in the overall population expressing the target in ≥5% and ≥25% of cells, in two independent trials (EMERGE and METRIC), suggesting that the mere expression of the target could not be sufficient to elicit effective and durable responses^168,169^. Furthermore, ladiratuzumab-vedotin, an ADC against the membrane zinc transporter protein LIV-1, is under investigation in several clinical trials (Table 1). Preliminary results have been observed with the combination of ladiratuzumab-vedotin and pembrolizumab in heavily pretreated TNBC patients, with 54% of ORR^170^.

Lately, the implementation of novel ADCs targeting HER2 provided encouraging results even in BC population with low levels of HER2 expression and no detectable ERBB2 amplifications, potentially defining a new role for HER2 also in TNBC. Trastuzumab-deruxtecan is an ADC carrying a topoisomerase I inhibitor payload that has been tested across several tumor types with varying levels of IHC HER2 expression. Recent data reported from a phase Ib trial showed an interesting activity of trastuzumab-deruxtecan in HER2-low tumors, though only a minority (13%) was TNBC. In the overall population, the study reported an ORR of 37.0% and mPFS of 11.7 months^171^, establishing HER2-low as a potential biomarker of patient selection in clinical trials with anti-HER2 ADCs.

### Mechanisms of resistance to standard therapies in TNBC

#### Resistance to chemotherapy

Chemotherapy currently represents the mainstay for TNBC treatment^172^. As aforementioned, molecular profiling can identify TNBC subtypes more likely to benefit from NAC^21,22^. However, TNBCs usually become resistant during treatments or can be intrinsically unresponsive to chemotherapy, with numerous possible mechanisms of chemoresistance.

One of the major mechanisms is mediated by ATP-binding cassette (ABC) transporters that causes the ATP-dependent efflux of various chemotherapy compounds across cellular membranes. Interestingly, a significantly higher expression or upregulation of multidrug-resistant protein-1 (ABCC1/MRP1), breast cancer resistance protein (ABCG2/BCRP) and multidrug-resistant protein-8 (ABCC11/MRP8) was observed in TNBC^173–176^. Each of these confers resistance to different agents, with extensively overlap. Several strategies aiming to inhibit activity or expression of the ABC transporters are being studying to overcome chemoresistance, as the use of sulindac, PZ-39 and various TKIs combined with chemotherapy^177–180^. However, many issues still exist, including the need to inhibit multiple transporters and the unacceptable associated toxicities.

The chemoresistance observed in TNBC may also be due to the presence of cancer stem cells (CSCs), a tumor cell subpopulation with the ability of self-renewal following treatment, subsequently leading to tumor re-growth^181^. Although CSCs have been observed in all BC subtypes, TNBC resulted to be intrinsically enriched in CSCs^182,183^. Moreover, accumulation of chemo-resistant CSCs has been described in residual tumor after NAC^184^. CSC-associated chemoresistance may be due to several factors including their relatively low proliferation rate and the high expression of ABC transporters^185^. Different therapeutic strategies to overcome CSCs chemoresistance are under evaluation, including targeting of CSC surface antigens and signaling pathways crucial for CSC self-renewal^181^.

Hypoxia is another described mechanism promoting tumor growth, survival, and therapy resistance^186^. Hypoxia alters TME and compromises uptake and/or activity of many cytotoxic agents, and confers resistance to radiation therapy. Moreover, hypoxia induces breast CSCs phenotype and promotes immunosuppression^187,188^. TNBC subtype is usually associated to high levels of hypoxia^189^. Use of hypoxia-activated prodrugs acting as cytotoxins^190^ or inhibition of molecular targets critical for hypoxia processes, including hypoxia-inducible factor inhibitors^191,192^, may potentially target hypoxia.

TP53 mutations, present in >50% of TNBC^28–32^, can also determine resistance to chemotherapy, especially platinum compounds^193^. As demonstrated in preclinical models, loss of p53 function promotes tolerance to platinum-induced DNA interstrand cross-links and double-strand breaks via G1 checkpoint abrogation and subsequent activation of alternative DNA-repair pathways that allows cancer cell survival^194^. The impact of TP53 mutation on response to specific chemotherapeutics is not well established in TNBC^195,196^, deserving further investigation. Furthermore, TNBC is encompassed by a complex network of different signaling pathways, including nuclear factor kappa-light-chain-enhancer of activated B cells (NF-kB), PTEN/PI3K/AKT/mTOR, and JAK/STAT, that promote TNBC progression and chemoresistance^4^. NF-kB is frequently upregulated in TNBC, while attempts to inhibit this pathway provided only modest results due to high toxicity^197,198^. PAM pathway is also frequently hyperactivated in TNBC, mainly due to PTEN loss, and is associated with poor prognosis and chemoresistance^199,200^. Similarly, abnormal JAK/STAT signaling is frequently observed in TNBC and a crosstalk between STAT3 and upregulation of ABC transporters have been observed^28,33^. However, the JAK1/2 inhibitor ruxolitinib did not demonstrate compelling efficacy as single-agent in a phase II trial^201^, corroborating the concept that inhibition of a single pathway is poorly effective in TNBC. Other clinical trials testing specific inhibitors of the JAK/STAT pathway are currently ongoing. Lastly, EGFR and insulin-like growth factor-1 receptor (IGF-1R) pathways are also implicated in TNBC chemoresistance^202–204^. Despite EGFR and IGF-1R overexpression in ∼40% of TNBCs^205,206^, disappointing results in clinical trials targeting these pathways have been obtained so far^76–81,206,207^.

#### Resistance to PARP inhibitors

Likewise other targeted therapies, acquired resistance to PARPis might develop over time through multiple different mechanisms^208^. One of the most well described is the restoration of sufficient HRR despite PARP inhibition, as in the case of secondary “revertant” mutations in BRCA1 or BRCA2^209–212^. Restoration of BRCA1 or BRCA2 protein functionality may occur by genetic events that may restore the open reading frame, thus leading to a functional protein, or by genetic reversion of the inherited mutation that may restore the wild-type protein. These events frequently determine resistance to platinum-based chemotherapy and PARPis^213^. Moreover, HRR may also be restored by loss of BRCA1 promoter methylation, leading to expression of BRCA1 gene similar to the one of HR-proficient tumors. Probably, this is due to a positive selection of tumor cells clones with lower promoter methylation^208^.

The ability to protect the stalled replication fork, despite HR defects, may represent another resistance mechanism^214^. Upregulation of the ATR/CHK1 pathway has been observed with the subsequent phosphorylation of multiple proteins contributing to fork stability^215^. Fork protection may also be obtained by reducing recruitment of the nucleases to the stalled replication fork, especially through the downregulation of the activity of different proteins, as PTIP and EZH2, responsible for nucleases recruitment. As a result, tumor cells may achieve resistance to PARPis without restoring HR^215^. Furthermore, since PARPi activity is mediated by the inhibition of PARP enzymes, another possible mechanism of resistance is represented by the decreased expression of these proteins^216^. Moreover, mutations of PARP1 and other proteins involved in DNA repair may be responsible for primary and secondary resistance to PARPis, respectively^217^. In addition, specific mutations of the BRCA1 and 2 genes, especially missense mutations that disrupts the N-terminal domain of BRCA1, may favor rapid resistance to PARPis. Mutation in the BRCA C terminal domain may generate protein products unable to fold properly, thus being more subject to protease-mediated degradation. Heat shock protein-90 (HSP90) stabilizes these mutant BRCA1 proteins that can in turn efficiently interact with PALB2-BRCA2-RAD51 complex, conferring PARPis and cisplatin resistance^218^. In this case, treatment with an HSP90 inhibitor may potentially restore cancer cell sensitivity to PARP inhibition.

Lastly, mutations in genes encoding for shieldin subunit complex^219,220^ and loss of REV7^221^ can cause resistance to PARPis in BRCA1-deficient cells, owing to restoration of HRR. Of note, some of these alterations in BRCA-deficient tumors confer sensitivity to platinum compounds, suggesting that the underlying mechanism of resistance could influence the TNBC therapeutic algorithm. Alternative treatment strategies that may revert or delay the emergence of resistant clones, including combining PARPis with other agents or targeting alternative molecules involved in DNA-damage response (ATR, ATM, WEE1), as well as the identification of the individual mechanism of resistance are required to identify potential therapeutic strategies to overcome resistance. Since some of these mechanisms may provide resistance also to other drugs, therapies given prior to and after PARPis should be carefully selected.

#### Resistance to immunotherapy

Primary and acquired resistance to ICIs have been observed in several patients. Understanding the multifactorial mechanisms of resistance to ICIs should go in parallel with the identification of reproducible and reliable biomarkers that may predict the likelihood and extent of response^222^.

Defective tumor immunorecognition may impair not only physiologic but also therapy-stimulated immunosurveillance. Indeed, an altered/insufficient antigen presentation or a limited neoantigen repertoire may contribute to dampen immune responses. Moreover, abundance and activation of CD8-postive T cells is key. Several strategies investigating the possible combination of multiple DNA damaging agents (chemotherapy, radiotherapy) or agents targeting DNA repair (PARP and ATR inhibitors) are being studied^223^. These agents can promote the release of neoantigens within the TME enhancing immunogenicity of the tumor and T-cell infiltration.

TME composition in influencing response to ICIs has been extensively studied, ranging from “immune-inflamed” to “immune-desert” phenotypes^224^. As aforementioned, high levels of TILs have been associated with better prognosis and promising response to immunotherapy in TNBC^123–126,159^. However, different subpopulations of T cells may have a different significance^225–227^. Regulatory T cells (CD4+/CD25high/FoxP3+) may promote tumor growth while inhibiting activity of cytotoxic CD8-positive T cells through direct cell-cell contact and/or secretion of transforming growth factor-β. The role of B lymphocytes is still not clear; activated B cells may participate in anti-tumor immune response through different mechanisms, including secretion of antigen-specific antibodies, induction of innate immune cells (e.g., M1 tumor-associated macrophages), release of different cytokines (e.g., IL-6) and activation of complement cascades. Recently, Kim et al. ^228^ classified TNBC in two “subtypes” according to neutrophils and macrophages infiltration, each with its own regulation pathways: neutrophil-enriched (NES), characterized by immunosuppressive neutrophils resistant to ICI, and macrophage-enriched subtype (MES) containing predominantly CCR2-dependent macrophages and exhibiting variable responses to ICI. Authors postulated that a MES-to-NES conversion may mediate acquired ICB resistance^228^. Furthermore, angiogenesis plays a crucial role in the formation of spatially-limited “immunoresistant niches”, difficult to reach by immune cells. The use of anti-angiogenic therapies may promote vascular normalization, improve lymphocyte trafficking across the endothelium and reverse immunotherapy resistance^229^.

Recent research has been focused on enteric microbiome as a potential factor influencing immunotherapy efficacy^230^. However, the impact of gut bacteria on response or resistance to ICIs in BC has not been elucidated yet. Potential strategies to manipulate gut microbiota are still under investigation to enhance anticancer therapy efficacy. Lastly, PTEN alterations have been found to be associated with reduced survival in TNBC patients treated with anti-PD-1/L1 ICIs^147^. As PAM pathway inhibition can revert the T-cell-mediated resistance^231^, a phase I trial is investigating the combination of ipatasertib, atezolizumab and paclitaxel in TNBC patients with promising antitumor activity (ORR 73%), regardless of PD-L1 expression and PIK3CA/AKT1/PTEN status^232^.

## Conclusions and future perspectives

TNBC is a heterogeneous disease that encompasses different histological and molecular subtypes with distinct outcomes. Each subtype is defined by specific transcriptomic and genetic alterations, which can be potentially targeted. However, only a small proportion of TNBC patients is actually treated with biomarker-driven therapies, as PARPis or platinum agents in germline BRCA1/2 carriers and ICIs in PD-L1-positive TNBCs. Therefore, chemotherapy remains the cornerstone of treatment for the largest part of patients. GEP allows to identify TNBC subtypes with distinct responses to neoadjuvant chemotherapy, supporting the implementation of standard and new therapies in selected TNBC subgroups. Not all TNBCs have a bad prognosis and, thus, risk stratification is necessary for tailoring escalation/de-escalation of therapies. Accordingly, recognizing histological and molecular heterogeneity of TNBC is essential to tailor treatment on individual basis to maximize the clinical benefit. However, several challenges have to be addressed.

The low frequency of highly recurrently mutated genes and potentially actionable alterations represent a relevant issue for the development of new therapeutic strategies. Whole-genome sequencing technologies can expand the number of patients amenable to targeted therapies, as hypothesized for patients with large-scale state transition-high or somatic biallelic loss-of-function in HR-related genes^31,233,234^. Furthermore, metastatic TNBC seems to display great heterogeneity and genetic complexity as compared to early stage^31^. Such observation suggests that tailored treatments should be implemented in the early setting to prevent the onset of complex and redundant mechanisms of resistance. In addition, multiple alterations can affect the same pathway at same time. For instance, TNBCs carrying PIK3CA activating mutations can concomitantly present PTEN loss thus not benefiting from PIK3CA-selective inhibition, as demonstrated in ER-positive BC^235,236^. Furthermore, new methodologies, such as liquid biopsy-based assays capable to identify tumors at very early stages and to monitor minimal residual disease, would allow to better predict outcomes during the disease course. Accordingly, new drugs could potentially be placed when disease is less complex biologically, identifying patients with breast cancer who need experimental therapy early in the disease course and for which fast-track drug approval is required.

The immune TME in TNBC considerably influences the risk of relapse and response to chemotherapy, providing the rationale for the application of ICI-based therapies. However, controversial results with immunotherapy in TNBC have been reported so far. Therefore, refinement of biomarker-selected TNBC population more likely to derive benefit from immunotherapy-based therapies represents the issue of several ongoing researches. The abovementioned biomarkers should not be interpreted as interchangeable, but complementary, since each one describes a feature of the complex cancer–immune interplay. In this way, a better definition of a TNBC “immunogram” is essential to properly select patients for immunotherapy-based treatments and, concomitantly, implement innovative strategies to enhance immunogenicity in “immune-excluded” cancers through combinatorial approaches.

In conclusion, TNBC is solely an operational term, considering the marked histopathological, transcriptomic and genomic heterogeneity that encompasses this BC subtype. The application of multi-omics technologies and biocomputational algorithms as well as novel clinical trial designs are strongly warranted to expand the therapeutic armamentarium against TNBC and pave the way towards personalized anticancer treatments.

## Data Availability

Source data for all tables are provided in the paper. Figures was generated by reanalysis of publicly available studies and open-source platforms using RStudio (version 1.1.463). No new datasets have been generated or analyzed for this article.

## References

[1] Foulkes WD, Smith IE, Reis-Filho JS (2010). Triple-negative breast cancer. N. Engl. J. Med..

[2] Perou CM (2000). Molecular portraits of human breast tumours. Nature.

[3] Rakha EA, Reis-Filho JS, Ellis IO (2008). Basal-like breast cancer: a critical review. J. Clin. Oncol..

[4] Garrido-Castro AC, Lin NU, Polyak K (2019). Insights into molecular classifications of triple-negative breast cancer: improving patient selection for treatment. Cancer Discov..

[5] Bertucci F (2008). How basal are triple-negative breast cancers?. Int. J. Cancer.

[6] Parker JS (2009). Supervised risk predictor of breast cancer based on intrinsic subtypes. J. Clin. Oncol..

[7] de Ronde JJ (2010). Concordance of clinical and molecular breast cancer subtyping in the context of preoperative chemotherapy response. Breast Cancer Res. Treat..

[8] Bastien RR (2012). PAM50 breast cancer subtyping by RT-qPCR and concordance with standard clinical molecular markers. BMC Med Genom..

[9] Prat A (2010). Phenotypic and molecular characterization of the claudin-low intrinsic subtype of breast cancer. Breast Cancer Res..

[10] Fougner C, Bergholtz H, Norum JH, Sorlie T (2020). Re-definition of claudin-low as a breast cancer phenotype. Nat. Commun..

[11] Fulford LG (2006). Specific morphological features predictive for the basal phenotype in grade 3 invasive ductal carcinoma of breast. Histopathology.

[12] Livasy CA (2006). Phenotypic evaluation of the basal-like subtype of invasive breast carcinoma. Mod. Pathol..

[13] Turner NC (2007). BRCA1 dysfunction in sporadic basal-like breast cancer. Oncogene.

[14] Kreike B (2007). Gene expression profiling and histopathological characterization of triple-negative/basal-like breast carcinomas. Breast Cancer Res..

[15] Sorlie T (2001). Gene expression patterns of breast carcinomas distinguish tumor subclasses with clinical implications. Proc. Natl Acad. Sci. USA.

[16] Sorlie T (2003). Repeated observation of breast tumor subtypes in independent gene expression data sets. Proc. Natl Acad. Sci. USA.

[17] Geyer FC (2017). The spectrum of triple-negative breast disease: high- and low-grade lesions. Am. J. Pathol..

[18] Lae M (2009). Secretory breast carcinomas with ETV6-NTRK3 fusion gene belong to the basal-like carcinoma spectrum. Mod. Pathol..

[19] Persson M (2009). Recurrent fusion of MYB and NFIB transcription factor genes in carcinomas of the breast and head and neck. Proc. Natl Acad. Sci. USA.

[20] Lehmann BD (2011). Identification of human triple-negative breast cancer subtypes and preclinical models for selection of targeted therapies. J. Clin. Invest..

[21] Masuda H (2013). Differential response to neoadjuvant chemotherapy among 7 triple-negative breast cancer molecular subtypes. Clin. Cancer Res..

[22] Lehmann BD (2016). Refinement of triple-negative breast cancer molecular subtypes: implications for neoadjuvant chemotherapy selection. PLoS ONE.

[23] Burstein MD (2015). Comprehensive genomic analysis identifies novel subtypes and targets of triple-negative breast cancer. Clin. Cancer Res..

[24] Curtis C (2012). The genomic and transcriptomic architecture of 2,000 breast tumours reveals novel subgroups. Nature.

[25] Jiang YZ (2019). Genomic and transcriptomic landscape of triple-negative breast cancers: subtypes and treatment strategies. Cancer Cell.

[26] Karaayvaz M (2018). Unravelling subclonal heterogeneity and aggressive disease states in TNBC through single-cell RNA-seq. Nat. Commun..

[27] Kim C (2018). Chemoresistance evolution in triple-negative breast cancer delineated by single-cell sequencing. Cell.

[28] Cancer Genome Atlas, N. (2012). Comprehensive molecular portraits of human breast tumours. Nature.

[29] Kandoth C (2013). Mutational landscape and significance across 12 major cancer types. Nature.

[30] Nik-Zainal S (2016). Landscape of somatic mutations in 560 breast cancer whole-genome sequences. Nature.

[31] Bertucci F (2019). Genomic characterization of metastatic breast cancers. Nature.

[32] Shah SP (2012). The clonal and mutational evolution spectrum of primary triple-negative breast cancers. Nature.

[33] Balko JM (2014). Molecular profiling of the residual disease of triple-negative breast cancers after neoadjuvant chemotherapy identifies actionable therapeutic targets. Cancer Discov..

[34] Bareche Y (2018). Unravelling triple-negative breast cancer molecular heterogeneity using an integrative multiomic analysis. Ann. Oncol..

[35] Tseng LM (2017). A comparison of the molecular subtypes of triple-negative breast cancer among non-Asian and Taiwanese women. Breast Cancer Res. Treat..

[36] Nik-Zainal S (2012). Mutational processes molding the genomes of 21 breast cancers. Cell.

[37] Alexandrov LB (2013). Signatures of mutational processes in human cancer. Nature.

[38] Alexandrov LB (2020). The repertoire of mutational signatures in human cancer. Nature.

[39] Swanton C, McGranahan N, Starrett GJ, Harris RS (2015). APOBEC enzymes: mutagenic fuel for cancer evolution and heterogeneity. Cancer Discov..

[40] Harris RS (2015). Molecular mechanism and clinical impact of APOBEC3B-catalyzed mutagenesis in breast cancer. Breast Cancer Res..

[41] Shang M (2018). Potential management of circulating tumor DNA as a biomarker in triple-negative breast cancer. J. Cancer.

[42] Wan JCM (2017). Liquid biopsies come of age: towards implementation of circulating tumour DNA. Nat. Rev. Cancer.

[43] Garcia-Murillas I (2015). Mutation tracking in circulating tumor DNA predicts relapse in early breast cancer. Sci. Transl. Med..

[44] Olsson E (2015). Serial monitoring of circulating tumor DNA in patients with primary breast cancer for detection of occult metastatic disease. EMBO Mol. Med..

[45] Radovich M (2020). Association of circulating tumor DNA and circulating tumor cells after neoadjuvant chemotherapy with disease recurrence in patients with triple-negative breast cancer: preplanned secondary analysis of the BRE12-158 randomized clinical trial. JAMA Oncol..

[46] Takeshita T (2015). Prognostic role of PIK3CA mutations of cell-free DNA in early-stage triple negative breast cancer. Cancer Sci..

[47] Madic J (2015). Circulating tumor DNA and circulating tumor cells in metastatic triple negative breast cancer patients. Int. J. Cancer.

[48] Stover DG (2018). Association of cell-free DNA tumor fraction and somatic copy number alterations with survival in metastatic triple-negative breast cancer. J. Clin. Oncol..

[49] Hilborn E (2016). Androgen receptor expression predicts beneficial tamoxifen response in oestrogen receptor-alpha-negative breast cancer. Br. J. Cancer.

[50] Xu M (2020). Prognostic significance of androgen receptor expression in triple negative breast cancer: a systematic review and meta-analysis. Clin. Breast Cancer.

[51] Di Zazzo E (2016). Prostate cancer stem cells: the role of androgen and estrogen receptors. Oncotarget.

[52] Basile D (2017). Androgen receptor in estrogen receptor positive breast cancer: beyond expression. Cancer Treat. Rev..

[53] Gucalp A (2013). Phase II trial of bicalutamide in patients with androgen receptor-positive, estrogen receptor-negative metastatic breast cancer. Clin. Cancer Res..

[54] Traina TA (2018). Enzalutamide for the treatment of androgen receptor-expressing triple-negative breast cancer. J. Clin. Oncol..

[55] Traina TA (2017). Overall survival (OS) in patients (Pts) with diagnostic positive (Dx+) breast cancer: Subgroup analysis from a phase 2 study of enzalutamide (ENZA), an androgen receptor (AR) inhibitor, in AR+ triple-negative breast cancer (TNBC) treated with 0-1 prior lines of therapy. J. Clin. Oncol..

[56] Bonnefoi H (2016). A phase II trial of abiraterone acetate plus prednisone in patients with triple-negative androgen receptor positive locally advanced or metastatic breast cancer (UCBG 12-1). Ann. Oncol..

[57] Marra A, Curigliano G (2019). Are all cyclin-dependent kinases 4/6 inhibitors created equal?. NPJ Breast Cancer.

[58] Vasan N, Toska E, Scaltriti M (2019). Overview of the relevance of PI3K pathway in HR-positive breast cancer. Ann. Oncol..

[59] Asghar US (2017). Single-cell dynamics determines response to CDK4/6 inhibition in triple-negative breast cancer. Clin. Cancer Res..

[60] Lehmann BD (2014). PIK3CA mutations in androgen receptor-positive triple negative breast cancer confer sensitivity to the combination of PI3K and androgen receptor inhibitors. Breast Cancer Res..

[61] Millis SZ (2015). Predictive biomarker profiling of >6000 breast cancer patients shows heterogeneity in TNBC, with treatment implications. Clin. Breast Cancer.

[62] Porta C, Paglino C, Mosca A (2014). Targeting PI3K/Akt/mTOR signaling in cancer. Front. Oncol..

[63] Mosele F (2020). Outcome and molecular landscape of patients with PIK3CA-mutated metastatic breast cancer. Ann. Oncol..

[64] Jovanovic B (2017). A randomized phase II neoadjuvant study of cisplatin, paclitaxel with or without everolimus in patients with stage II/III triple-negative breast cancer (TNBC): responses and long-term outcome correlated with increased frequency of DNA damage response gene mutations, TNBC subtype, AR status, and Ki67. Clin. Cancer Res..

[65] Martin M (2017). A randomized adaptive phase II/III study of buparlisib, a pan-class I PI3K inhibitor, combined with paclitaxel for the treatment of HER2- advanced breast cancer (BELLE-4). Ann. Oncol..

[66] Sharma P (2018). Clinical and biomarker results from phase I/II study of PI3K inhibitor BYL 719 (alpelisib) plus nab-paclitaxel in HER2-negative metastatic breast cancer. J. Clin. Oncol..

[67] Kim SB (2017). Ipatasertib plus paclitaxel versus placebo plus paclitaxel as first-line therapy for metastatic triple-negative breast cancer (LOTUS): a multicentre, randomised, double-blind, placebo-controlled, phase 2 trial. Lancet Oncol..

[68] Dent R (2018). Overall survival (OS) update of the double-blind placebo (PBO)-controlled randomized phase 2 LOTUS trial of first-line ipatasertib (IPAT) + paclitaxel (PAC) for locally advanced/metastatic triple-negative breast cancer (mTNBC). J. Clin. Oncol..

[69] Schmid P (2020). Capivasertib plus paclitaxel versus placebo plus paclitaxel as first-line therapy for metastatic triple-negative breast cancer: the PAKT trial. J. Clin. Oncol..

[70] Fruman DA (2017). The PI3K pathway in human disease. Cell.

[71] Hopkins BD (2018). Suppression of insulin feedback enhances the efficacy of PI3K inhibitors. Nature.

[72] Vernieri C (2016). Targeting cancer metabolism: dietary and pharmacologic interventions. Cancer Discov..

[73] Zhang W, Liu HT (2002). MAPK signal pathways in the regulation of cell proliferation in mammalian cells. Cell Res..

[74] Balko JM (2012). Profiling of residual breast cancers after neoadjuvant chemotherapy identifies DUSP4 deficiency as a mechanism of drug resistance. Nat. Med..

[75] Duncan JS (2012). Dynamic reprogramming of the kinome in response to targeted MEK inhibition in triple-negative breast cancer. Cell.

[76] Di Leo A (2008). Phase III, double-blind, randomized study comparing lapatinib plus paclitaxel with placebo plus paclitaxel as first-line treatment for metastatic breast cancer. J. Clin. Oncol..

[77] Finn RS (2009). Estrogen receptor, progesterone receptor, human epidermal growth factor receptor 2 (HER2), and epidermal growth factor receptor expression and benefit from lapatinib in a randomized trial of paclitaxel with lapatinib or placebo as first-line treatment in HER2-negative or unknown metastatic breast cancer. J. Clin. Oncol..

[78] Baselga J (2013). Randomized phase II study of the anti-epidermal growth factor receptor monoclonal antibody cetuximab with cisplatin versus cisplatin alone in patients with metastatic triple-negative breast cancer. J. Clin. Oncol..

[79] Nabholtz JM (2016). Multicentric neoadjuvant pilot Phase II study of cetuximab combined with docetaxel in operable triple negative breast cancer. Int. J. Cancer.

[80] Yardley DA (2016). Panitumumab, gemcitabine, and carboplatin as treatment for women with metastatic triple-negative breast cancer: a sarah cannon research institute phase II trial. Clin. Breast Cancer.

[81] Matsuda N (2018). Safety and efficacy of panitumumab plus neoadjuvant chemotherapy in patients with primary HER2-negative inflammatory breast cancer. JAMA Oncol..

[82] Costa R (2017). Targeting epidermal growth factor receptor in triple negative breast cancer: new discoveries and practical insights for drug development. Cancer Treat. Rev..

[83] Ali R, Wendt MK (2017). The paradoxical functions of EGFR during breast cancer progression. Signal Transduct Target Ther..

[84] Hyman DM (2018). HER kinase inhibition in patients with HER2- and HER3-mutant cancers. Nature.

[85] Brufsky A (2018). Abstract P5-21-01: Cobimetinib combined with paclitaxel as first-line treatment for patients with advanced triple-negative breast cancer (COLET study): primary analysis of cohort I. Cancer Res..

[86] Wongchenko M (2016). Exploratory biomarker analysis of first-line cobimetinib (C) + paclitaxel (P) in patients (pts) with advanced triple-negative breast cancer (TNBC) from the phase 2 COLET study. Eur. J. Cancer.

[87] Brufsky A (2019). Phase II COLET study: atezolizumab (A) + cobimetinib (C) + paclitaxel (P)/nab-paclitaxel (nP) as first-line (1L) treatment (tx) for patients (pts) with locally advanced or metastatic triple-negative breast cancer (mTNBC). J. Clin. Oncol..

[88] Schafer JM (2020). Targeting MYCN-expressing triple-negative breast cancer with BET and MEK inhibitors. Sci. Transl. Med..

[89] Albanell J (2016). 228PD - BRAF genomic alterations in breast cancer. Ann. Oncol..

[90] Hyman DM (2015). Vemurafenib in multiple nonmelanoma cancers with BRAF V600 mutations. N. Engl. J. Med..

[91] Subbiah V (2020). Pan-cancer efficacy of vemurafenib in BRAFV600-mutant non-melanoma cancers. Cancer Discov..

[92] Jing J (2012). Comprehensive predictive biomarker analysis for MEK inhibitor GSK1120212. Mol. Cancer Ther..

[93] Liu Y, Shepherd EG, Nelin LD (2007). MAPK phosphatases–regulating the immune response. Nat. Rev. Immunol..

[94] Livraghi L, Garber JE (2015). PARP inhibitors in the management of breast cancer: current data and future prospects. BMC Med..

[95] Chen S, Parmigiani G (2007). Meta-analysis of BRCA1 and BRCA2 penetrance. J. Clin. Oncol..

[96] Goodwin PJ (2012). Breast cancer prognosis in BRCA1 and BRCA2 mutation carriers: an International Prospective Breast Cancer Family Registry population-based cohort study. J. Clin. Oncol..

[97] Rebbeck TR (2015). Association of type and location of BRCA1 and BRCA2 mutations with risk of breast and ovarian cancer. JAMA.

[98] Foulkes WD (2003). Germline BRCA1 mutations and a basal epithelial phenotype in breast cancer. J. Natl Cancer Inst..

[99] Lord CJ, Ashworth A (2016). BRCAness revisited. Nat. Rev. Cancer.

[100] Farmer H (2005). Targeting the DNA repair defect in BRCA mutant cells as a therapeutic strategy. Nature.

[101] O’Neil NJ, Bailey ML, Hieter P (2017). Synthetic lethality and cancer. Nat. Rev. Genet..

[102] Davies H (2017). HRDetect is a predictor of BRCA1 and BRCA2 deficiency based on mutational signatures. Nat. Med..

[103] Robson M (2017). Olaparib for metastatic breast cancer in patients with a germline BRCA mutation. N. Engl. J. Med..

[104] Litton JK (2018). Talazoparib in patients with advanced breast cancer and a germline BRCA mutation. N. Engl. J. Med..

[105] Han HS (2018). Veliparib with temozolomide or carboplatin/paclitaxel versus placebo with carboplatin/paclitaxel in patients with BRCA1/2 locally recurrent/metastatic breast cancer: randomized phase II study. Ann. Oncol..

[106] Dieras V (2020). Veliparib with carboplatin and paclitaxel in BRCA-mutated advanced breast cancer (BROCADE3): a randomised, double-blind, placebo-controlled, phase 3 trial. Lancet Oncol..

[107] Tutt A (2018). Carboplatin in BRCA1/2-mutated and triple-negative breast cancer BRCAness subgroups: the TNT trial. Nat. Med..

[108] Tung N (2020). TBCRC 031: randomized phase II study of neoadjuvant cisplatin versus doxorubicin-cyclophosphamide in germline BRCA carriers with HER2-negative breast cancer (the INFORM trial). J. Clin. Oncol..

[109] Fasching PA (2019). GeparOLA: A randomized phase II trial to assess the efficacy of paclitaxel and olaparib in comparison to paclitaxel/carboplatin followed by epirubicin/cyclophosphamide as neoadjuvant chemotherapy in patients (pts) with HER2-negative early breast cancer (BC) and homologous recombination deficiency (HRD). J. Clin. Oncol..

[110] O’Shaughnessy J (2014). Phase III study of iniparib plus gemcitabine and carboplatin versus gemcitabine and carboplatin in patients with metastatic triple-negative breast cancer. J. Clin. Oncol..

[111] Loibl S (2018). Addition of the PARP inhibitor veliparib plus carboplatin or carboplatin alone to standard neoadjuvant chemotherapy in triple-negative breast cancer (BrighTNess): a randomised, phase 3 trial. Lancet Oncol..

[112] Falchook G (2019). Alisertib in combination with weekly paclitaxel in patients with advanced breast cancer or recurrent ovarian cancer: a randomized clinical trial. JAMA Oncol..

[113] Diamond JR (2018). A phase II clinical trial of the Aurora and angiogenic kinase inhibitor ENMD-2076 for previously treated, advanced, or metastatic triple-negative breast cancer. Breast Cancer Res..

[114] Telli ML (2018). Abstract OT2-07-07: ATR inhibitor M6620 (formerly VX-970) with cisplatin in metastatic triple-negative breast cancer: Preliminary results from a phase 1 dose expansion cohort (NCT02157792). Cancer Res..

[115] Infante JR (2017). Phase I study of GDC-0425, a checkpoint kinase 1 inhibitor, in combination with gemcitabine in patients with refractory solid tumors. Clin. Cancer Res..

[116] Ibrahim YH (2012). PI3K inhibition impairs BRCA1/2 expression and sensitizes BRCA-proficient triple-negative breast cancer to PARP inhibition. Cancer Discov..

[117] Matulonis UA (2017). Phase I dose escalation study of the PI3kinase pathway inhibitor BKM120 and the oral poly (ADP ribose) polymerase (PARP) inhibitor olaparib for the treatment of high-grade serous ovarian and breast cancer. Ann. Oncol..

[118] Jiao S (2017). PARP inhibitor upregulates PD-L1 expression and enhances cancer-associated immunosuppression. Clin. Cancer Res..

[119] Mouw KW, Goldberg MS, Konstantinopoulos PA, D’Andrea AD (2017). DNA damage and repair biomarkers of immunotherapy response. Cancer Disco..

[120] Sato H (2017). DNA double-strand break repair pathway regulates PD-L1 expression in cancer cells. Nat. Commun..

[121] Lee JM (2017). Safety and clinical activity of the programmed death-ligand 1 inhibitor durvalumab in combination with poly (ADP-Ribose) polymerase inhibitor olaparib or vascular endothelial growth factor receptor 1-3 inhibitor cediranib in women’s cancers: a dose-escalation, phase I study. J. Clin. Oncol..

[122] Vinayak S (2019). Open-label clinical trial of niraparib combined with pembrolizumab for treatment of advanced or metastatic triple-negative breast cancer. JAMA Oncol..

[123] Loi S (2019). Tumor-infiltrating lymphocytes and prognosis: a pooled individual patient analysis of early-stage triple-negative breast cancers. J. Clin. Oncol..

[124] Denkert C (2018). Tumour-infiltrating lymphocytes and prognosis in different subtypes of breast cancer: a pooled analysis of 3771 patients treated with neoadjuvant therapy. Lancet Oncol..

[125] Pruneri G (2016). Clinical validity of tumor-infiltrating lymphocytes analysis in patients with triple-negative breast cancer. Ann. Oncol..

[126] Park JH (2019). Prognostic value of tumor-infiltrating lymphocytes in patients with early-stage triple-negative breast cancers (TNBC) who did not receive adjuvant chemotherapy. Ann. Oncol..

[127] Nanda R (2016). Pembrolizumab in patients with advanced triple-negative breast cancer: phase Ib KEYNOTE-012 study. J. Clin. Oncol..

[128] Adams S (2019). Pembrolizumab monotherapy for previously treated metastatic triple-negative breast cancer: cohort A of the phase II KEYNOTE-086 study. Ann. Oncol..

[129] Adams S (2019). Pembrolizumab monotherapy for previously untreated, PD-L1-positive, metastatic triple-negative breast cancer: cohort B of the phase II KEYNOTE-086 study. Ann. Oncol..

[130] Cortés J (2019). LBA21 - KEYNOTE-119: Phase III study of pembrolizumab (pembro) versus single-agent chemotherapy (chemo) for metastatic triple negative breast cancer (mTNBC). Ann. Oncol..

[131] Dirix LY (2018). Avelumab, an anti-PD-L1 antibody, in patients with locally advanced or metastatic breast cancer: a phase 1b JAVELIN Solid Tumor study. Breast Cancer Res. Treat..

[132] Emens LA (2019). Long-term clinical outcomes and biomarker analyses of atezolizumab therapy for patients with metastatic triple-negative breast cancer: a phase 1 study. JAMA Oncol..

[133] Adams S (2019). Atezolizumab plus nab-paclitaxel in the treatment of metastatic triple-negative breast cancer with 2-year survival follow-up: a phase 1b clinical trial. JAMA Oncol..

[134] Tolaney SM (2020). A phase Ib/II study of eribulin (ERI) plus pembrolizumab (PEMBRO) in metastatic triple-negative breast cancer (mTNBC) (ENHANCE 1). J. Clin. Oncol..

[135] Schmid P (2018). Atezolizumab and Nab-paclitaxel in advanced triple-negative breast cancer. N. Engl. J. Med..

[136] Schmid P (2020). Atezolizumab plus nab-paclitaxel as first-line treatment for unresectable, locally advanced or metastatic triple-negative breast cancer (IMpassion130): updated efficacy results from a randomised, double-blind, placebo-controlled, phase 3 trial. Lancet Oncol..

[137] Rugo HS (2019). LBA20-Performance of PD-L1 immunohistochemistry (IHC) assays in unresectable locally advanced or metastatic triple-negative breast cancer (mTNBC): post-hoc analysis of IMpassion130. Ann. Oncol..

[138] Ribas A, Hu-Lieskovan S (2016). What does PD-L1 positive or negative mean?. J. Exp. Med..

[139] Schmid P (2020). Pembrolizumab for early triple-negative breast cancer. N. Engl. J. Med..

[140] Schmid P (2019). LBA8_PR - KEYNOTE-522: phase III study of pembrolizumab (pembro) + chemotherapy (chemo) vs placebo (pbo) + chemo as neoadjuvant treatment, followed by pembro vs pbo as adjuvant treatment for early triple-negative breast cancer (TNBC). Ann. Oncol..

[141] Gianni L (2020). Abstract GS3-04: Pathologic complete response (pCR) to neoadjuvant treatment with or without atezolizumab in triple negative, early high-risk and locally advanced breast cancer. NeoTRIPaPDL1 Michelangelo randomized study. Cancer Res..

[142] Voorwerk L (2019). Immune induction strategies in metastatic triple-negative breast cancer to enhance the sensitivity to PD-1 blockade: the TONIC trial. Nat. Med..

[143] Marra A, Viale G, Curigliano G (2019). Recent advances in triple negative breast cancer: the immunotherapy era. BMC Med..

[144] Samstein RM (2019). Tumor mutational load predicts survival after immunotherapy across multiple cancer types. Nat. Genet..

[145] Yarchoan M, Hopkins A, Jaffee EM (2017). Tumor Mutational Burden and Response Rate to PD-1 Inhibition. N. Engl. J. Med..

[146] Barroso-Sousa R (2020). Prevalence and mutational determinants of high tumor mutation burden in breast cancer. Ann. Oncol..

[147] Barroso-Sousa R (2020). Tumor mutational burden and PTEN alterations as molecular correlates of response to PD-1/L1 blockade in metastatic triple-negative breast cancer. Clin Cancer Res..

[148] McGranahan N, Swanton C (2019). Neoantigen quality, not quantity. Sci. Transl. Med..

[149] Luksza M (2017). A neoantigen fitness model predicts tumour response to checkpoint blockade immunotherapy. Nature.

[150] Criscitiello C (2018). A gene signature to predict high tumor-infiltrating lymphocytes after neoadjuvant chemotherapy and outcome in patients with triple-negative breast cancer. Ann. Oncol..

[151] Hendrickx W (2017). Identification of genetic determinants of breast cancer immune phenotypes by integrative genome-scale analysis. Oncoimmunology.

[152] Sharma P (2019). Validation of the DNA damage immune response signature in patients with triple-negative breast cancer from the SWOG 9313c trial. J. Clin. Oncol..

[153] McGrail DJ (2018). Multi-omics analysis reveals neoantigen-independent immune cell infiltration in copy-number driven cancers. Nat. Commun..

[154] Havel JJ, Chowell D, Chan TA (2019). The evolving landscape of biomarkers for checkpoint inhibitor immunotherapy. Nat. Rev. Cancer.

[155] Loi S (2017). LBA13Relationship between tumor infiltrating lymphocyte (TIL) levels and response to pembrolizumab (pembro) in metastatic triple-negative breast cancer (mTNBC): results from KEYNOTE-086. Ann. Oncol..

[156] Schmid P (2020). Pembrolizumab plus chemotherapy as neoadjuvant treatment of high-risk, early-stage triple-negative breast cancer: results from the phase 1b open-label, multicohort KEYNOTE-173 study. Ann. Oncol..

[157] Emens LA (2019). Abstract GS1-04: IMpassion130: efficacy in immune biomarker subgroups from the global, randomized, double-blind, placebo-controlled, phase III study of atezolizumab + nab-paclitaxel in patients with treatment-naïve, locally advanced or metastatic triple-negative breast cancer. Cancer Res..

[158] Loi S (2020). Abstract PD5-03: relationship between tumor-infiltrating lymphocytes (TILs) and outcomes in the KEYNOTE-119 study of pembrolizumab vs chemotherapy for previously treated metastatic triple-negative breast cancer (mTNBC). Cancer Res..

[159] Byrne A (2020). Tissue-resident memory T cells in breast cancer control and immunotherapy responses. Nat. Rev. Clin. Oncol..

[160] Sade-Feldman M (2019). Defining T cell states associated with response to checkpoint immunotherapy in melanoma. Cell.

[161] Coats S (2019). Antibody-drug conjugates: future directions in clinical and translational strategies to improve the therapeutic index. Clin. Cancer Res..

[162] Sharkey RM (2015). Enhanced delivery of SN-38 to human tumor xenografts with an anti-trop-2-SN-38 antibody conjugate (Sacituzumab Govitecan). Clin. Cancer Res..

[163] Trerotola M (2013). Upregulation of Trop-2 quantitatively stimulates human cancer growth. Oncogene.

[164] Huang H (2005). Aberrant expression of novel and previously described cell membrane markers in human breast cancer cell lines and tumors. Clin. Cancer Res..

[165] Bardia A (2019). Sacituzumab Govitecan-hziy in refractory metastatic triple-negative breast cancer. N. Engl. J. Med..

[166] Immunomedics announces ASCENT study to be stopped for compelling efficacy [news release]. Morris Plains, New York, Immunomedics; 6 Apr 2020.

[167] Rose AA (2010). Glycoprotein nonmetastatic B is an independent prognostic indicator of recurrence and a novel therapeutic target in breast cancer. Clin. Cancer Res..

[168] Yardley DA (2015). EMERGE: a randomized phase II study of the antibody-drug conjugate glembatumumab vedotin in advanced glycoprotein NMB-expressing breast cancer. J. Clin. Oncol..

[169] Vahdat, L. T. et al. Abstract P6-20-01: METRIC: a randomized international phase 2b study of the antibody-drug conjugate (ADC) glembatumumab vedotin (GV) in gpNMB-overexpressing, metastatic, triple-negative breast cancer (mTNBC). *Cancer Res.* **79**, P6-20-01 (2019).

[170] Han H (2020). Abstract PD1-06: open label phase 1b/2 study of ladiratuzumab vedotin in combination with pembrolizumab for first-line treatment of patients with unresectable locally-advanced or metastatic triple-negative breast cancer. Cancer Res..

[171] Modi S (2020). Antitumor activity and safety of trastuzumab deruxtecan in patients with HER2-low-expressing advanced breast cancer: results from a phase Ib study. J. Clin. Oncol..

[172] Bianchini G, Balko JM, Mayer IA, Sanders ME, Gianni L (2016). Triple-negative breast cancer: challenges and opportunities of a heterogeneous disease. Nat. Rev. Clin. Oncol..

[173] Yamada A (2013). High expression of ATP-binding cassette transporter ABCC11 in breast tumors is associated with aggressive subtypes and low disease-free survival. Breast Cancer Res. Treat..

[174] Mahmood NA, Abdulghany ZS, Al-Sudani IM (2018). Expression of aldehyde dehydrogenase (ALDH1) and ATP Binding Cassette Transporter G2 (ABCG2) in Iraqi patients with colon cancer and the relation with clinicopathological features. Int. J. Mol. Cell Med..

[175] Arumugam A (2019). Silencing growth hormone receptor inhibits estrogen receptor negative breast cancer through ATP-binding cassette sub-family G member 2. Exp. Mol. Med..

[176] Guestini F (2019). Impact of Topoisomerase IIalpha, PTEN, ABCC1/MRP1, and KI67 on triple-negative breast cancer patients treated with neoadjuvant chemotherapy. Breast Cancer Res. Treat..

[177] O’Connor R (2007). A phase I clinical and pharmacokinetic study of the multi-drug resistance protein-1 (MRP-1) inhibitor sulindac, in combination with epirubicin in patients with advanced cancer. Cancer Chemother. Pharm..

[178] Peng H (2009). A novel two mode-acting inhibitor of ABCG2-mediated multidrug transport and resistance in cancer chemotherapy. PLoS ONE.

[179] Wu S, Fu L (2018). Tyrosine kinase inhibitors enhanced the efficacy of conventional chemotherapeutic agent in multidrug resistant cancer cells. Mol. Cancer.

[180] Wang S (2017). Indomethacin-based stimuli-responsive micelles combined with paclitaxel to overcome multidrug resistance. Oncotarget.

[181] Clara JA, Monge C, Yang Y, Takebe N (2020). Targeting signalling pathways and the immune microenvironment of cancer stem cells - a clinical update. Nat. Rev. Clin. Oncol..

[182] Park SY (2010). Heterogeneity for stem cell-related markers according to tumor subtype and histologic stage in breast cancer. Clin. Cancer Res..

[183] Ma F (2014). Enriched CD44(+)/CD24(-) population drives the aggressive phenotypes presented in triple-negative breast cancer (TNBC). Cancer Lett..

[184] Lee HE (2011). An increase in cancer stem cell population after primary systemic therapy is a poor prognostic factor in breast cancer. Br. J. Cancer.

[185] Zhou S (2001). The ABC transporter Bcrp1/ABCG2 is expressed in a wide variety of stem cells and is a molecular determinant of the side-population phenotype. Nat. Med..

[186] Vaupel P (2008). Hypoxia and aggressive tumor phenotype: implications for therapy and prognosis. Oncologist.

[187] Cosse JP, Michiels C (2008). Tumour hypoxia affects the responsiveness of cancer cells to chemotherapy and promotes cancer progression. Anticancer Agents Med. Chem..

[188] Lv Y (2015). Hypoxia-inducible factor-1alpha induces multidrug resistance protein in colon cancer. Oncol. Targets Ther..

[189] Tan EY (2009). The key hypoxia regulated gene CAIX is upregulated in basal-like breast tumours and is associated with resistance to chemotherapy. Br. J. Cancer.

[190] Wilson WR, Hay MP (2011). Targeting hypoxia in cancer therapy. Nat. Rev. Cancer.

[191] Ban HS (2017). The novel hypoxia-inducible factor-1alpha inhibitor IDF-11774 regulates cancer metabolism, thereby suppressing tumor growth. Cell Death Dis..

[192] Soni S, Padwad YS (2017). HIF-1 in cancer therapy: two decade long story of a transcription factor. Acta Oncol..

[193] Friboulet L, Soria JC, Olaussen KA (2019). The “Guardian of the Genome”-An Old Key to Unlock the ERCC1 Issue. Clin. Cancer Res..

[194] Heyza JR (2019). Identification and characterization of synthetic viability with ERCC1 deficiency in response to interstrand crosslinks in lung cancer. Clin. Cancer Res..

[195] Isakoff SJ (2015). TBCRC009: a multicenter phase II clinical trial of platinum monotherapy with biomarker assessment in metastatic triple-negative breast cancer. J. Clin. Oncol..

[196] Bae SY (2018). Differences in prognosis and efficacy of chemotherapy by p53 expression in triple-negative breast cancer. Breast Cancer Res. Treat..

[197] Begalli, F. et al. Unlocking the NF-kappaB conundrum: embracing complexity to achieve specificity. *Biomedicines* **5**, 50 (2017). 10.3390/biomedicines5030050 PMC5618308 28829404

[198] Messeha SS (2018). The inhibitory effects of plumbagin on the NF-B pathway and CCL2 release in racially different triple-negative breast cancer cells. PLoS ONE.

[199] Ueng SH (2012). Phosphorylated mTOR expression correlates with poor outcome in early-stage triple negative breast carcinomas. Int. J. Clin. Exp. Pathol..

[200] Steelman LS (2008). Suppression of PTEN function increases breast cancer chemotherapeutic drug resistance while conferring sensitivity to mTOR inhibitors. Oncogene.

[201] Stover DG (2018). Phase II study of ruxolitinib, a selective JAK1/2 inhibitor, in patients with metastatic triple-negative breast cancer. NPJ Breast Cancer.

[202] Kim A, Jang MH, Lee SJ, Bae YK (2017). Mutations of the epidermal growth factor receptor gene in triple-negative breast cancer. J. Breast Cancer.

[203] Park HS (2014). High EGFR gene copy number predicts poor outcome in triple-negative breast cancer. Mod. Pathol..

[204] Farabaugh SM, Boone DN, Lee AV (2015). Role of IGF1R in breast cancer subtypes, stemness, and lineage differentiation. Front Endocrinol..

[205] Masuda H (2012). Role of epidermal growth factor receptor in breast cancer. Breast Cancer Res. Treat..

[206] Ekyalongo, R. C. & Yee, D. Revisiting the IGF-1R as a breast cancer target. *NPJ Precis. Oncol.* **1**, 1–7 (2017). 10.1038/s41698-017-0017-y PMC5687252 29152592

[207] Yee D (2012). Insulin-like growth factor receptor inhibitors: baby or the bathwater?. J. Natl Cancer Inst..

[208] D’Andrea AD (2018). Mechanisms of PARP inhibitor sensitivity and resistance. DNA Repair.

[209] Sakai W (2008). Secondary mutations as a mechanism of cisplatin resistance in BRCA2-mutated cancers. Nature.

[210] Norquist B (2011). Secondary somatic mutations restoring BRCA1/2 predict chemotherapy resistance in hereditary ovarian carcinomas. J. Clin. Oncol..

[211] Barber LJ (2013). Secondary mutations in BRCA2 associated with clinical resistance to a PARP inhibitor. J. Pathol..

[212] Waks AG (2020). Reversion and non-reversion mechanisms of resistance to PARP inhibitor or platinum chemotherapy in BRCA1/2-mutant metastatic breast cancer. Ann. Oncol..

[213] Quigley D (2017). Analysis of circulating cell-free DNA identifies multiclonal heterogeneity of BRCA2 reversion mutations associated with resistance to PARP inhibitors. Cancer Discov..

[214] Schlacher K, Wu H, Jasin M (2012). A distinct replication fork protection pathway connects Fanconi anemia tumor suppressors to RAD51-BRCA1/2. Cancer Cell.

[215] Ray Chaudhuri A (2016). Replication fork stability confers chemoresistance in BRCA-deficient cells. Nature.

[216] Sonnenblick A, de Azambuja E, Azim HA, Piccart M (2015). An update on PARP inhibitors–moving to the adjuvant setting. Nat. Rev. Clin. Oncol..

[217] Pettitt SJ (2018). Genome-wide and high-density CRISPR-Cas9 screens identify point mutations in PARP1 causing PARP inhibitor resistance. Nat. Commun..

[218] Johnson N (2013). Stabilization of mutant BRCA1 protein confers PARP inhibitor and platinum resistance. Proc. Natl Acad. Sci. USA.

[219] Noordermeer SM (2018). The shieldin complex mediates 53BP1-dependent DNA repair. Nature.

[220] Dev H (2018). Shieldin complex promotes DNA end-joining and counters homologous recombination in BRCA1-null cells. Nat. Cell Biol..

[221] Xu G (2015). REV7 counteracts DNA double-strand break resection and affects PARP inhibition. Nature.

[222] Syn NL, Teng MWL, Mok TSK, Soo RA (2017). De-novo and acquired resistance to immune checkpoint targeting. Lancet Oncol..

[223] Brown JS, Sundar R, Lopez J (2018). Combining DNA damaging therapeutics with immunotherapy: more haste, less speed. Br. J. Cancer.

[224] Chen DS, Mellman I (2017). Elements of cancer immunity and the cancer-immune set point. Nature.

[225] Nelson BH (2010). CD20+ B cells: the other tumor-infiltrating lymphocytes. J. Immunol..

[226] Rodriguez-Pinto D (2005). B cells as antigen presenting cells. Cell Immunol..

[227] DeNardo DG, Andreu P, Coussens LM (2010). Interactions between lymphocytes and myeloid cells regulate pro- versus anti-tumor immunity. Cancer Metastasis Rev..

[228] Kim IS (2019). Immuno-subtyping of breast cancer reveals distinct myeloid cell profiles and immunotherapy resistance mechanisms. Nat. Cell Biol..

[229] Li Q (2020). Low-dose anti-angiogenic therapy sensitizes breast cancer to PD-1 blockade. Clin. Cancer Res..

[230] Gopalakrishnan V, Helmink BA, Spencer CN, Reuben A, Wargo JA (2018). The influence of the gut microbiome on cancer, immunity, and cancer immunotherapy. Cancer Cell.

[231] Peng W (2016). Loss of PTEN promotes resistance to T cell-mediated immunotherapy. Cancer Discov..

[232] Schmid P (2019). Abstract CT049: phase Ib study evaluating a triplet combination of ipatasertib (IPAT), atezolizumab (atezo), and paclitaxel (PAC) or nab-PAC as first-line (1L) therapy for locally advanced/metastatic triple-negative breast cancer (TNBC). Cancer Res..

[233] Popova T (2012). Ploidy and large-scale genomic instability consistently identify basal-like breast carcinomas with BRCA1/2 inactivation. Cancer Res..

[234] Riaz N (2017). Pan-cancer analysis of bi-allelic alterations in homologous recombination DNA repair genes. Nat. Commun..

[235] Juric D (2015). Convergent loss of PTEN leads to clinical resistance to a PI(3)Kalpha inhibitor. Nature.

[236] Razavi P (2020). Alterations in PTEN and ESR1 promote clinical resistance to alpelisib plus aromatase inhibitors. Nat. Cancer.

